# Integrated cytokine and metabolite analysis reveals immunometabolic reprogramming in COVID-19 patients with therapeutic implications

**DOI:** 10.1038/s41467-021-21907-9

**Published:** 2021-03-12

**Authors:** Nan Xiao, Meng Nie, Huanhuan Pang, Bohong Wang, Jieli Hu, Xiangjun Meng, Ke Li, Xiaorong Ran, Quanxin Long, Haijun Deng, Na Chen, Shao Li, Ni Tang, Ailong Huang, Zeping Hu

**Affiliations:** 1 School of Pharmaceutical Sciences, Tsinghua University, Beijing, 100084 China; 2 Tsinghua-Peking Joint Center for Life Sciences, Tsinghua University, Beijing, 100084 China; 3 Key Laboratory of Molecular Biology for Infectious Diseases (Ministry of Education), Chongqing Medical University, Chongqing, China; 4 NHC Key Laboratory of Biotechnology of Antibiotics, Institute of Medicinal Biotechnology, Chinese Academy of Medical Sciences & Peking Union Medical College, Beijing, 100050 China; 5 Agilent Technologies (China), Chaoyang District, Beijing, 100102 China; 6 Institute for TCM-X, MOE Key Laboratory of Bioinformatics, Bioinformatics Division, BNRIST, Department of Automation, Tsinghua University, Beijing, 100084 China; 7 Beijing Frontier Research Center for Biological Structure, Tsinghua University, Beijing, 100084 China

**Keywords:** Metabolomics, Cytokines, SARS-CoV-2, Prognostic markers

## Abstract

Cytokine release syndrome (CRS) is a major cause of the multi-organ injury and fatal outcome induced by SARS-CoV-2 infection in severe COVID-19 patients. Metabolism can modulate the immune responses against infectious diseases, yet our understanding remains limited on how host metabolism correlates with inflammatory responses and affects cytokine release in COVID-19 patients. Here we perform both metabolomics and cytokine/chemokine profiling on serum samples from healthy controls, mild and severe COVID-19 patients, and delineate their global metabolic and immune response landscape. Correlation analyses show tight associations between metabolites and proinflammatory cytokines/chemokines, such as IL-6, M-CSF, IL-1α, IL-1β, and imply a potential regulatory crosstalk between arginine, tryptophan, purine metabolism and hyperinflammation. Importantly, we also demonstrate that targeting metabolism markedly modulates the proinflammatory cytokines release by peripheral blood mononuclear cells isolated from SARS-CoV-2-infected rhesus macaques ex vivo, hinting that exploiting metabolic alterations may be a potential strategy for treating fatal CRS in COVID-19.

## Abstract (web summary)

Metabolism changes can modulate immune responses in many contexts, and vice versa. Here the authors associate metabolomic, as well as cytokine and chemokine, data from stratified COVID-19 patients to find that arginine, tryptophan and purine metabolic pathways correlate with hyperproliferation, thus hinting at potential therapeutic targets for severe COVID-19 patients.

## Introduction

Coronavirus disease 2019 (COVID-19), caused by highly infectious severe acute respiratory syndrome coronavirus 2 (SARS-CoV-2), has recently become global pandemic^1^, highlighting an urgent need for effective therapeutic strategies. SARS-CoV-2 infection triggers immune response contributing to both virus clearance and acute respiratory distress syndrome (ARDS) development^2,3^. The severe COVID-19 patients often experience cytokine-release syndrome (CRS) referred to as “cytokine storm”, which is characterized by excessive proinflammatory cytokine release and leads to widespread damage, multiple organ failure, and fatal clinical outcomes^4–8^. Emerging clinical trials reveal that immunomodulatory drugs, such as the IL-6 receptor-blocking antibody tocilizumab and JAK1/2 inhibitor ruxolitinib^9–11^, can dampen the hyperactive immune response, suggesting cytokine-release blockade as a promising treatment option. Hence, identifying the key factors driving CRS induced by SARS-CoV-2 infection is of utmost importance to provide fresh insights for novel immunomodulatory therapies.

The association between metabolism and immunity has been reported since 1960s^12^. In recent years, many immunometabolism studies have further illustrated the interplay between host metabolism and immune responses^13^. For example, the immunoregulatory metabolite succinate, which is linked to the mitochondrial metabolism, has been recognized as the innate immune signal that enhances the IL-1β production during inflammation^14^. Reciprocally, infection-induced proinflammatory cytokines, such as IL-6, can modulate glucose and lipid metabolism, indicating the guiding role of cytokines in host metabolism reprogramming^15,16^. Interestingly, the metabolite itaconate, identified as an anti-inflammatory regulator of a set of cytokines (e.g., IL-6 and IL-12), is shown to be induced by type I interferons and conversely to limit the type I interferon responses, indicating a negative-feedback loop that involves itaconate and cytokines^17–19^. Notably, metabolic pathways have been reported to regulate the innate and adaptive host responses to the infection of various viruses, such as human immunodeficiency virus (HIV)^20^, yellow fever virus^21^, and severe fever with thrombocytopenia syndrome virus (SFTSV)^22^. In particular, glucose metabolism plays an important role in regulating influenza A virus-induced cytokine storm^23^. Since the COVID-19 pandemic, a few studies exploring the immunological and metabolic signatures in the patients have been reported^5,7,24–26^. The circulating serum metabolites and inflammatory cytokines have been shown to be tightly correlated in COVID-19 patients^26–28^. A recent study also reveals that elevated glucose levels in COVID-19 patients promote SARS-CoV-2 replication and cytokine production in monocytes^29^. However, our understanding on the host metabolism–immune response correlation landscape and, particularly, the potential modulatory role of the perturbed metabolism in inflammatory responses upon SARS-CoV-2 infection is still limited.

In this study, we characterize the globally dysregulated metabolic pathways and cytokine/chemokine levels in COVID-19 patients compared to healthy controls. We identify the escalated correlations between circulating metabolites and cytokines/chemokines from mild to severe patients, and further reveal the disturbed metabolic pathways linked to hyperinflammation in severe COVID-19. We also demonstrate that targeting arginine, tryptophan, or purine metabolism by metabolite supplementation or pharmacological inhibition modulates the ex vivo inflammatory cytokine release by isolated peripheral blood mononuclear cells (PBMCs) derived from SARS-CoV-2-infected rhesus macaques. Overall, this study provides novel insights into the immunometabolic interplay in COVID-19 patients and suggests that metabolic interventions may be potentially exploited as rational strategies to suppress SARS-CoV-2-induced CRS.

## Results

### Study design and patient cohort

**Fig. 1:**
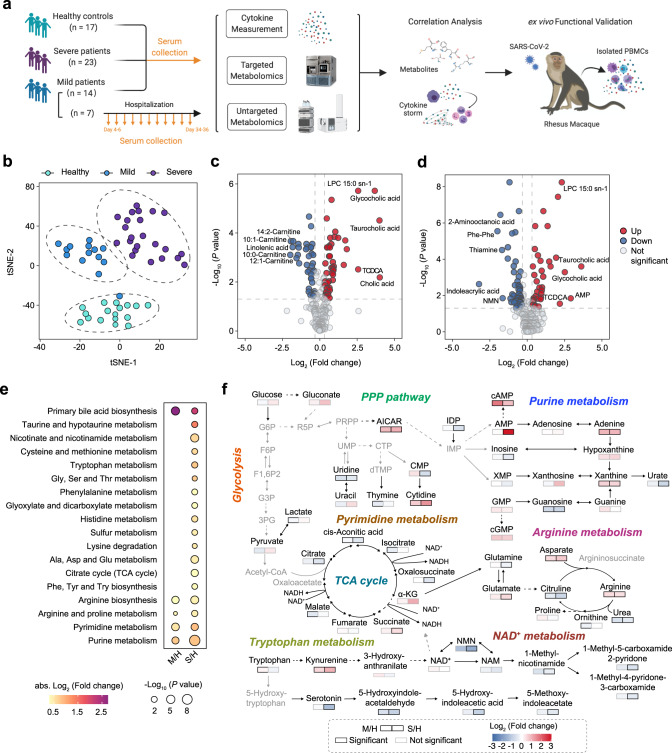
Study design and metabolic profiling in serum samples from mild and severe COVID-19 patients. **a** Overview of cohort (including 21 mild patients, 23 severe COVID-19 patients, and 17 healthy controls) and the study design. **b** *t*-SNE plot distributed healthy controls (*n* = 17), mild patients (*n* = 14), and severe patients (*n* = 23) according to serum metabolites detected from targeted and untargeted metabolomics. **c, d** Volcano plots comparing serum metabolites of mild (**c**) or severe (**d**) patients with healthy controls. Significantly altered metabolites are highlighted in red (increased) and blue (decreased). The top 5 metabolites that significantly increased or decreased are marked with text. Two-sided Mann-Whitney *U* test followed by Benjamini-Hochberg (BH) multiple comparison test with FDR < 0.05 and fold change >1.25 or <0.8. **e** KEGG metabolic pathways enriched by significantly changed serum metabolites in mild (**c**) and severe (**d**) patients. One-sided Fisher’s exact test followed by BH multiple comparison test with FDR < 0.1. **f** Schematic depicting the key disturbed metabolic pathways in response to SARS-CoV-2 infection. Gray nodes represent metabolites that were not tested. Metabolite alterations are represented by color intensity, and borders are color-coded by statistical significance.

To understand how the host metabolism correlates with CRS in COVID-19 patients, we performed both metabolomics and cytokine profiling on serum samples from the same symptomatic COVID-19 patient cohort. The cohort comprises 17 healthy controls, 14 mild, and 23 severe COVID-19 patients. The serum samples were taken from the patients that were about 1–18 days post admission to the hospital. An additional independent cohort of 7 mild patients with longitudinal follow-up time-points that occurred 4–36 days post symptom onset was also included. We collected the clinical information and conducted immunological and biochemical laboratory tests as well as metabolomics and cytokine profiling on the serum samples from these patients. To ascertain the association between metabolism and cytokine release, we performed correlation analysis between the levels of metabolites and cytokines, and further validated the functional effects of the metabolic intervention on cytokine release ex vivo by PBMCs derived from SARS-CoV-2-infected rhesus macaques (Fig. 1a). The basic clinical features of the cohort were detailed in Table S1. Significant reduction in lymphocyte count, and marked increase in C-reactive protein (CRP), alanine amino-transferase (ALT), aspartate aminotransferase (AST), direct bilirubin (DBIL), and glucose were exhibited in severe COVID-19 patients, which were consistent with previous findings^1,30^ (Supplementary Fig. 1 and Supplementary Data 1).

### Metabolic profiling of serum samples from COVID-19 patients

To determine the metabolic perturbations associated with SARS-CoV-2 infection, we profiled the serum samples from 17 healthy controls and 44 COVID-19 patients by using both targeted and untargeted metabolomics analyses (Supplementary Fig. 2a and Supplementary Data 2 and 3). Targeted metabolomics analysis was performed on an ultra-high-performance liquid chromatography–triple quadruple mass spectrometry system (UHPLC-MS/MS). A total of 258 metabolites were monitored and 134 metabolites were reliably detected by the targeted metabolomics method. Untargeted metabolomics analysis was performed on an UHPLC quadruple TOF high-resolution MS/MS system. After data preprocessing and metabolite identification, 155 metabolites were identified from 6072 metabolite features extracted from the raw data acquired in positive- and negative-ionization modes. A total of 36 metabolites were identified from both targeted and untargeted metabolomics and showed consistent alterations along with the increasing disease severity. By integrating the targeted and untargeted metabolomics datasets, we identified a total of 253 metabolites, including 134 metabolites (with the 36 overlap metabolites included) from targeted metabolic profiling and 119 metabolites (with the 36 overlap metabolites excluded) from untargeted metabolic profiling, and observed distinct metabolic profiles among healthy controls (*n* = 17), mild (*n* = 14), and severe (*n* = 23) patients (Fig. 1b and Supplementary Fig. 2b–d). To determine whether SARS-CoV-2 infection shares the same metabolic disturbances with other respiratory diseases, we collected serum samples from 20 non-COVID-19 acute upper respiratory tract infection patients followed by a targeted metabolomics analysis (Supplementary Data 2 and 3). A clear segregation among the three groups (i.e., healthy controls, COVID-19 patients, non-COVID-19 acute upper respiratory tract infection patients) was observed, suggesting the uniquely disturbed metabolic profiles in COVID-19 patients (Supplementary Fig. 2e). Volcano plots highlighted 89 differential metabolites (FDR < 0.05, fold change >1.25 or <0.8, 50 up and 39 down) between healthy controls (*n* = 17) and mild patients (*n* = 14), and 88 differential metabolites (FDR < 0.05, fold change >1.25 or <0.8, 37 up and 51 down) between healthy controls (*n* = 17) and severe patients (*n* = 23), reflecting markedly dysregulated metabolic status of COVID-19 (Fig. 1c, d).

To characterize the dysregulated metabolic pathways in COVID-19 patients compared to healthy controls, we performed pathway enrichment analyses and observed that primary bile acid biosynthesis, amino acid metabolism, and nucleic acid metabolism were significantly perturbated in both mild and severe patients (Fig. 1e). In contrast, several metabolic pathways, such as nicotinate and nicotinamide metabolism, tryptophan metabolism, and citrate cycle (TCA cycle) were altered only in severe patients (Fig. 1e). Interestingly, metabolites that displayed constant upward or downward trend along disease severity were mostly associated with purine metabolism, nicotinate and nicotinamide metabolism, tryptophan metabolism, TCA cycle, and arginine metabolism (Supplementary Fig. 2f). Intermediates in arginine metabolism have been regarded as regulators of lymphocyte suppression during immune response^31^. Our previous work has demonstrated that arginine deficiency is associated with T cell dysregulation in SFTSV infection^22^. Here we observed that glutamate and aspartic acid were upregulated, while glutamine and citrulline were downregulated along disease severity (Supplementary Fig. 2g). Succinate, an intermediate of TCA cycle that has been proved to be an innate immune signaling molecule during inflammation in the macrophage^14^, displayed a successive increase along disease severity (Supplementary Fig. 2h). Moreover, the increase in kynurenine and decline in tryptophan and serotonin suggested the enhanced activity of the rate-limiting enzyme indole 2,3-dioxygenase 1 (IDO1) (Supplementary Fig. 2i), which is reported to be a modulator of inflammation^32,33^. In addition, NAD^+^ metabolism is found to be altered by host–pathogen interactions during innate and adaptive immune responses^34^, including SARS-COV-2 infection^35^. We observed that the level of nicotinamide mononucleotide (NMN), a key metabolite in the NAD^+^ metabolism, decreased as the severity of COVID-19 increases (Supplementary Fig. 2i). Of note, these core disturbed metabolites in COVID-19 patients showed no significant changes in non-COVID-19 acute upper respiratory tract infection patients when compared to healthy controls (Supplementary Fig. 2f–i). We also observed that several lysophosphatidylcholines (LPCs) were increased in COVID-19 patients (Supplementary Fig. 2j). Taken together, our data delineate the global metabolic alterations along the increase in the severity of COVID-19 (Fig. 1f).

### Cytokines release correlates with altered metabolism in COVID-19 patients

Extensive studies proved that metabolism and immune response are inextricably linked^13,20,36^. To clarify the correlation between metabolism and inflammatory responses after SARS-CoV-2 infection, we assessed the cytokine and chemokine levels in COVID-19 patients and healthy controls (Supplementary Data 4). Consistent with previous study reports^5–8,37^, COVID-19 patients presented marked elevation (FDR < 0.05) in 28 cytokines and chemokines compared to healthy controls (Supplementary Fig. 3a). Particularly, the CRS-related cytokines, including IL-6, IL-1β, IL-10, IL-18, and IFN-γ, displayed progressive increase along disease severity from healthy controls to mild and severe patients (Supplementary Fig. 3b), highlighting the broad and strong inflammatory responses in severe COVID-19 patients.

**Fig. 2:**
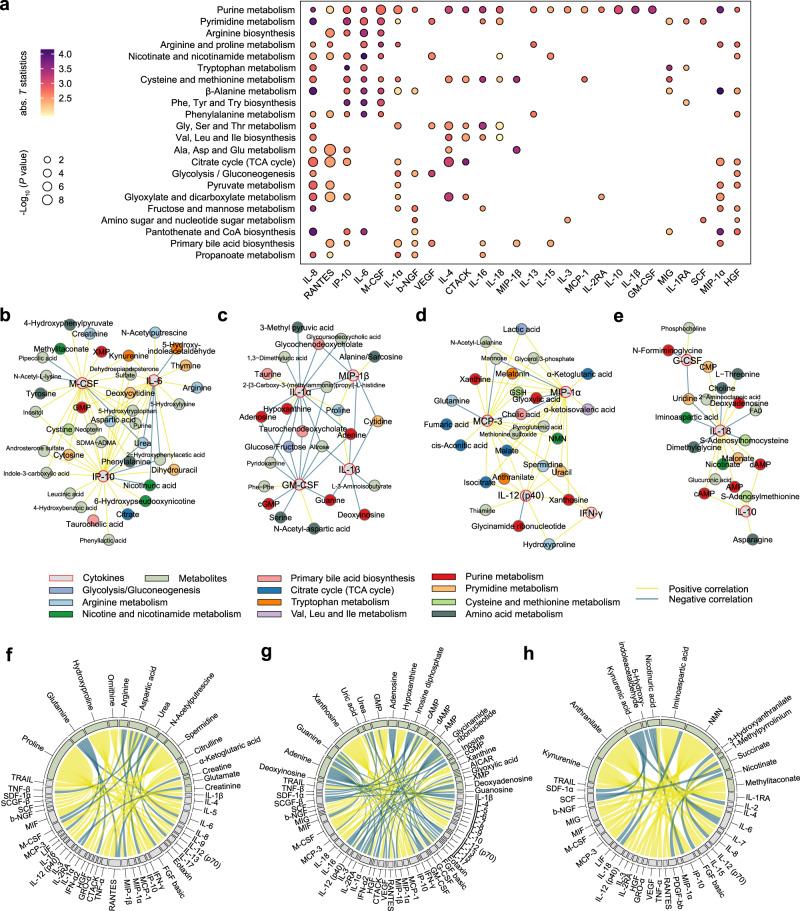
Metabolite–cytokine correlation in serum samples from COVID-19 patients. **a** Pathway enrichment analysis of metabolites significantly associated with the indicated cytokines in severe patients (*n* = 23). Two-sided *t* test followed by Benjamini-Hochberg (BH) multiple comparison test with FDR < 0.1. “abs. *T* statistics” is the mean absolute *T* statistics of significant metabolites in the pathway and is represented by color intensity. The dot size represents pathway significance (one-sided Fisher’s exact test followed by BH multiple comparison test). **b**–**e** Correlation networks of key CRS-related cytokines and metabolites in severe patients. Nodes and edges are color-coded by molecule types and metabolic pathways, and association directions, respectively. Networks were clustered by fast greedy modularity optimization algorithm. **f**–**h** Chord diagrams depicting the significant correlations of cytokines with metabolites involved in arginine metabolism (**f**), purine metabolism (**g**), tryptophan and NAD^+^ metabolism (**h**), respectively, in severe patients. Chords are color-coded by association directions consistent with (**b**–**e**).

Next, using linear regression after adjusting for age and gender (Eq. (1), Methods), we performed independent correlation analysis between cytokines and metabolites in mild and severe patients, respectively. Cytokine–metabolite correlations with FDR < 0.1 were considered significant. Systematic pathway analysis revealed that the dysregulated metabolic pathways highly correlated with several cytokines (e.g., IL-15, IL-10, and IL-2RA) in mild patients (Supplementary Fig. 4a). However, a tight correlation between dysregulated metabolic pathways and important inflammatory cytokines (e.g., IL-6, IP-10, IL-8, M-CSF, and IL-1α) in severe patients was observed (Fig. 2a). Of note, the inflammatory cytokines, including IL-6, IP-10, and M-CSF, showed increasing correlations with metabolites from mild to severe patients (Supplementary Fig. 4b–d). Specifically, 8 and 20 correlations were observed between IL-6 and metabolites in mild and severe patients, respectively; 10 and 33 correlations were observed between IP-10 and metabolites in mild and severe patients, respectively; 6 and 29 correlations were observed between M-CSF and metabolites in mild and severe patients, respectively (Supplementary Fig. 4b–d). In addition, the general correlations between metabolites and cytokines were intensified in severe patients compared to those in mild patients (Supplementary Fig. 4e, f). Next, we focused on the correlations between metabolites and inflammatory cytokines linked to CRS in severe patients. Interestingly, 14 inflammatory cytokines, such as IL-6, M-CSF, IP-10, GM-CSF, IL-18, IL-1α, and IL-1β, strongly correlated with metabolites involved in arginine metabolism, tryptophan and NAD^+^ metabolism, purine and pyrimidine metabolism, cysteine and methionine metabolism, TCA cycle, and primary bile acid metabolism in severe patients (Fig. 2b–e). These observations provide evidence that host metabolic reprogramming broadly and highly correlates with inflammatory cytokines linked to CRS. Notably, several key metabolites involved in arginine metabolism (e.g., arginine, glutamine, and aspartic acid, citrulline, urea, and proline) displayed strong correlation with CRS-related cytokines (e.g., IL-6, IL-1β, M-CSF, IL-12 p70, IFN-α2) in severe patients (Fig. 2f). In addition, purine metabolism (e.g., xanthosine, xanthine, guanosine, adenine, GMP, adenosine, and guanine) exhibited strong correlation with CRS-related cytokines (e.g., IL-6, M-CSF, MCP-3, GM-CSF, IL-1α, and IL-1β) (Fig. 2g). Metabolites involved in tryptophan and NAD^+^ metabolism, such as kynurenine and NMN, showed positive correlation with MCP-3, M-CSF, and IL-6. In contrast, nicotinic acid had negative correlation with IL-6 and IP-10 (Fig. 2h). Collectively, the correlations between metabolites and cytokines in severe patients suggest that the disturbances in these metabolic pathways are inextricably linked to the hyperinflammation in COVID-19.

### Longitudinal metabolite–cytokine correlation in follow-up mild COVID-19 patients

**Fig. 3:**
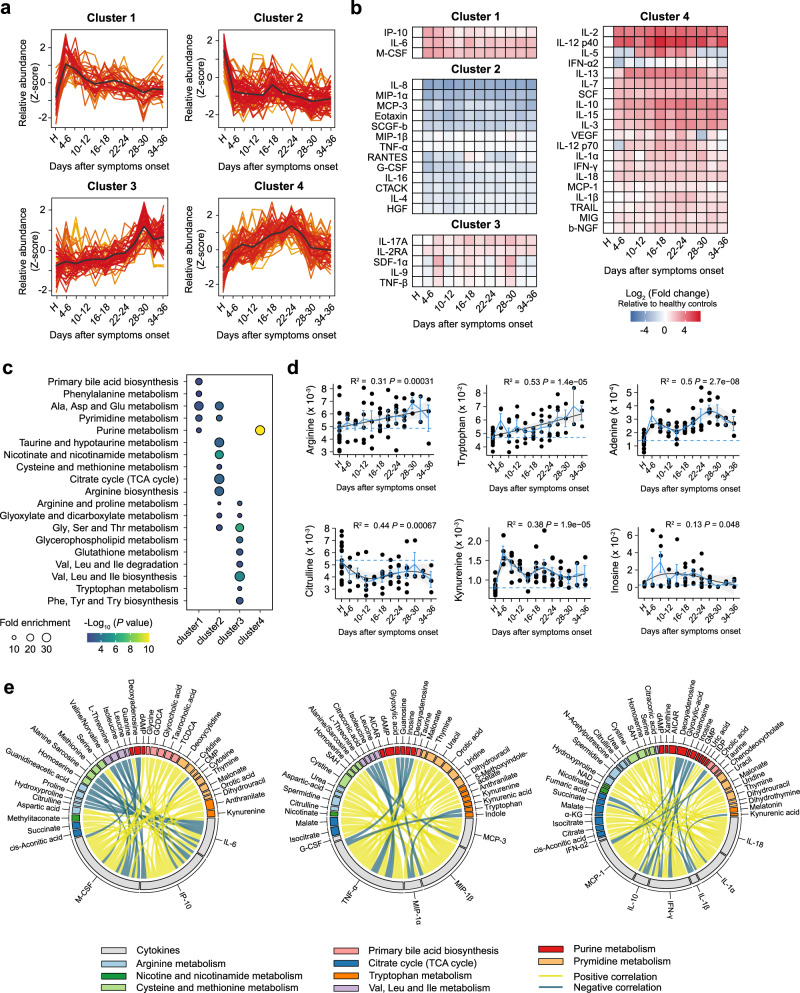
Longitudinal trajectories and metabolite–cytokine correlation in mild COVID-19 patients. **a** Longitudinal trajectory clustering of significantly changed serum metabolites, cytokines in follow-up patients (*n* = 7) with mild COVID-19. Metabolite and cytokine abundance in healthy controls were used as base line. Black lines represent the average trajectory for each cluster. **b** Heatmap comparison of cytokines at distinct time-points in follow-up patients (*n* = 7). Color intensity represents the Log2 fold change of mean cytokine abundance in each interval relative to healthy controls. **c** Pathway enrichment analysis of metabolites in each cluster. One-sided Fisher’s exact test followed by Benjamini-Hochberg (BH) multiple comparison test with FDR < 0.1. **d** Relative abundance trajectories of metabolites in follow-up patients (*n* = 7). Blue solid lines pass through the mean of each measurement at the specific time interval, and dotted lines represent the mean of measurements in healthy controls (*n* = 17). Generalized additive model (GAM) regression lines are represented by the black solid lines, with 95% confidence intervals for the regression line donated by gray filled areas. *P* value was assessed by one-way ANOVA. Data are presented as mean ± SEM. with individual data points shown. **e** Chord diagrams of significant associations between metabolites and core CRS-related cytokines in cluster 1 (left), cluster 2 (middle), and clusters 3 and 4 (right), respectively. Two-sided *t* test followed by BH multiple comparison test.

To further identify the dynamic correlations between metabolites and cytokines at longitudinal stages after SARS-CoV-2 infection, we performed *c*-means clustering analysis on both metabolite and cytokine data from the hospitalized mild patients at 4–36 days after symptom onset (Supplementary Data 2–4). We identified four main clusters of longitudinal trajectories that characterized distinct metabolic and immune signatures in these patients with acute antibody responses to SARS-CoV-2 infection^38^ (Supplementary Fig. 5a, b). Molecules enriched in cluster 1 increased at symptoms onset but gradually deceased during hospitalization; molecules in cluster 2 exhibited a sharp decrease at symptoms onset and sustained stable levels in later time-points; however, molecules in cluster 3 sustained steady levels but presented a delayed elevation in the very late events; cluster 4 contained molecules that elevated gradually and declined in late phases (Fig. 3a).

Three CRS-related cytokines including IL-6, IP-10, and M-CSF belonged to cluster 1 (Fig. 3b). Notably, the levels of IL-6, which is highly correlative to CRS^39^, slightly decreased during the first 2 weeks of symptom onset and remained at low level in later phases (Fig. 3b and Supplementary Fig. 5c). The IFN-γ-inducible protein, IP-10/CXCL-10, is considered as a member of CXC chemokine family with proinflammatory and severity-related properties in COVID-19^8^. The levels of IP-10 showed a sharp decline in the initial phase of treatment and sustained at relatively low levels during hospitalization (Fig. 3b and Supplementary Fig. 5c). Consistently, the myeloid cytokine M-CSF also decreased rapidly during the initial treatment phase and sustained a stable level afterwards (Fig. 3b and Supplementary Fig. 5c). In addition, proinflammatory cytokines in cluster 2, including G-CSF, IL-8, MIP-1α, and MCP-3, also showed decreased levels in mild patients compared to healthy controls and remained at steady levels during hospitalization (Fig. 3b and Supplementary Fig. 5c). However, proinflammatory cytokines in cluster 3 (e.g., IL-17A and TNF-β) and cluster 4 (e.g., IL-1α, IL-1β, IL-18, and MCP-1) showed the upward trend along the follow-up time-points (Fig. 3b and Supplementary Fig. 5c). These observations indicate that alleviation of inflammatory immune responses may be accompanied with clinical recovery in hospitalized mild patients.

Interestingly, several cytokines enriched in cluster 4 were associated with suppression of the inflammatory responses and viral replication, such as IL-10 and IFN-α2 (Fig. 3b and Supplementary Fig. 5c). IL-10, which reportedly plays a role in antagonizing inflammatory cell populations and suppressing immune hyperactivity^40^, continued to elevate over time and was maintained at high levels (Fig. 3b and Supplementary Fig. 5c). Also, IFN-α2 is reported to play a crucial role in combating infection through inhibiting viral replication and preventing viral entry into neighboring cells, thus used for treating several viral infections, including hepatitis B and C^41^. We observed the steadily elevated and sustained levels of IFN-α2 over hospitalization (Fig. 3b and Supplementary Fig. 5c). The increased levels of IL-10 and IFN-α2 may reflect the presence of a negative-feedback loop to control the inflammatory responses and virus infection. These data suggest that a protective immune response may occur along with downward trend of proinflammatory cytokines and clinical recovery in hospitalized mild patients^42^.

We next characterized metabolites that were enriched in four clusters and specific metabolite–cytokine correlations (Eq. (1), Methods). Interestingly, metabolites associated with arginine metabolism were enriched in clusters 2 and 3 (Fig. 3c). We observed an upward trend of arginine, ornithine, glutamate, and proline, whereas a decrease in citrulline (Fig. 3d and Supplementary Fig. 6a, b). Arginine metabolism reportedly played a crucial role in the regulation of immune responses^22,31^. Correlation analysis showed that intermediates in arginine metabolism highly correlated with proinflammatory cytokines including IL-6, M-CSF, and MIP-1β (Fig. 3e). Dysregulated tryptophan metabolism and NAD^+^ metabolism were evident by a marked alteration in associated metabolites, which exhibited four trajectories (Fig. 3c). The increased metabolites (i.e., tryptophan, indole) and decreased metabolites (i.e., kynurenine, kynurenic acid) reflected the attenuation of the hyperactivation of tryptophan-kynurenine pathway (Fig. 3d and Supplementary Fig. 6a, b), which plays an important role in modulating the inflammation^32^. Moreover, kynurenine positively correlated with proinflammatory cytokines including IP-10, MCP-3, and M-CSF, whereas tryptophan negatively correlated with MIP-1α, suggesting the regulatory role of tryptophan metabolism in inflammatory responses (Fig. 3e). Additionally, a large proportion of intermediates in purine metabolism displayed the upward trend, such as inosine and adenine (Fig. 3d and Supplementary Fig. 6a, b), and showed the negative correlation with most proinflammatory cytokines (Fig. 3e). Overall, our data identify the longitudinal metabolite–cytokine correlation dynamics along with clinical recovery of hospitalized mild patients.

### Effects of modulating metabolism on cytokine release by PBMCs ex vivo

**Fig. 4:**
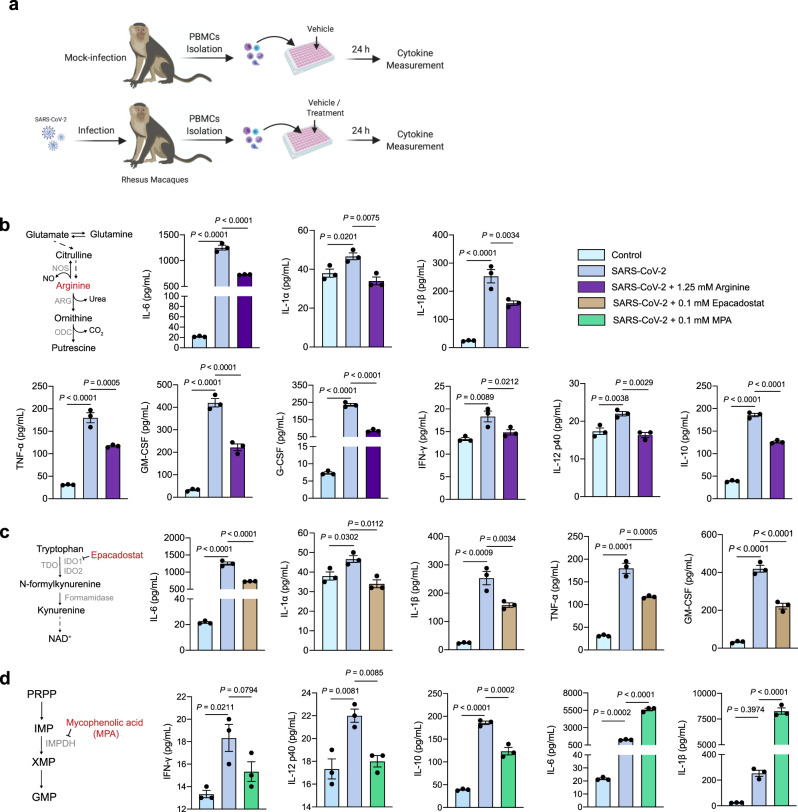
Targeting metabolism modulates cytokine release in PBMCs ex vivo model. **a** Schematic representation of the experimental workflow. PBMCs, isolated from peripheral blood of the mock-infected and SARS-CoV-2-infected rhesus macaques, were seeded in 96-well plates with vehicle or different drugs dissolved in medium; 24 h post-seeding, cytokine abundance in cell culture was quantified. **b**–**d** Metabolism diagrams and level of indicated cytokines and chemokines measured 24 h after supplementation of 1.25 mM arginine (**b**), 0.1 mM IDO1 inhibitor Epacadostat (**c**), and 0.1 mM inosine monophosphate dehydrogenase (IMPDH) inhibitor mycophenolic acid (MPA, **d**) in PBMCs (*n* = 3). Data are presented as mean ± SEM. with individual data points shown. One-way ANOVA followed by Benjamini-Hochberg (BH) multiple comparison test.

Our data presented above delineated the strong correlation between cytokines and metabolites, we therefore asked whether intervening arginine metabolism, tryptophan metabolism, and purine metabolism could regulate cytokine induction. To this end, we first experimentally infected a rhesus macaque (female, 5-years old) with SARS-CoV-2^43^. Although no obvious clinical signs were observed during the infection course, the SARS-CoV-2-infected rhesus macaque indeed showed a slight decrease in body weight from 1 to 8 days post infection (d.p.i.) and 8% weight loss at 8 d.p.i. (Supplementary Fig. 7a, b). The viral RNA load in nasal and throat swab was detectable at 7 d.p.i. (Supplementary Fig. 7c). We also found the local inflammatory infiltration with thickening of the alveolar in lung tissues of the SARS-CoV-2-infected rhesus macaque (Supplementary Fig. 7d). In addition, some proinflammatory cytokines, including IL-6, MCP-1, IL-10, G-CSF, IL-12 (p40), MIP-1β, TGF-α, and IL-1α, showed obvious increase at 7 d.p.i. (Supplementary Fig. 7e). These results were consistent with previous studies showing rhesus macaques can be infected with SARS-CoV-2 and exhibit rapid virus replication and inflammatory response^44–47^. We then isolated PBMCs from the SARS-CoV-2-infected and mock-infected rhesus macaques at 7 d.p.i., and measured the cytokine level after treatment with metabolites or compounds interfering with these key metabolic pathways, by optimized concentrations (Fig. 4a and Supplementary Fig. 8).

Interestingly, we observed that supplementation of arginine markedly inhibited the SARS-CoV-2-induced proinflammatory cytokine release by PBMCs, most of which are linked to CRS including IL-1α, IL-1β, IL-2, IL-6, TNF-α, GM-CSF, G-CSF, and MIP-1α (Fig. 4b, Supplementary Fig. 9, and Supplementary Data 4). Notably, the elevated level of IL-10, which is responsible for the inhibition of proinflammatory cytokine release from macrophage and dendritic cell (DC) populations^40^, was also suppressed (Fig. 4b). However, arginine supplementation did not alter the cytokine release (Supplementary Fig. 10 and Supplementary Data 4) or metabolic profiles including arginine-related metabolites (Supplementary Fig. 11 and Supplementary Data 3) in PBMCs isolated from healthy donors. These observations suggest that serum arginine metabolism may play an ameliorative role in the SARS-CoV-2-induced hyperinflammation, which, thus, could be exploited as a potential therapeutic target for CRS in COVID-19.

The conversion of tryptophan into kynurenine in immune cells is finely regulated by the enzyme IDO1, which is reportedly involved in regulating hyperinflammatory responses^33^. The elevated ratio between circulating kynurenine and tryptophan (Kyn/Trp) in patient’s serum described above suggested an increased activity of IDO1. Addition of Epacadostat, an IDO1 inhibitor, suppressed the SARS-CoV-2-induced proinflammatory cytokine release including IL-1α, IL-1β, IL-6, TNF-α, GM-CSF, G-CSF, IL-17A, and MIP-1α, which confirmed an essential role of tryptophan metabolism in exaggerated cytokine release upon SARS-CoV-2 infection (Fig. 4c, Supplementary Fig. 12, and Supplementary Data 4). Notably, treatment with Epacadostat also significantly suppressed the baseline cytokine release, such as IL-6, IL-1α, and IL-1β, in PBMCs isolated from healthy donors (Supplementary Fig. 13 and Supplementary Data 4). In addition, direct inhibition of purine metabolism with mycophenolic acid (MPA), which blocks the rate-limiting enzyme inosine monophosphate dehydrogenase (IMPDH) in de novo synthesis of guanosine nucleotides, significantly reduced the levels of IL-10, IFN-γ, IL-15, IL-12 p40, IL-17A, and TNF-α induced by SARS-CoV-2 infection. However, a profound increase in proinflammatory cytokines of IL-6, GM-CSF, IL-1α, and IL-1β was also observed, which suggests the exacerbated hyperinflammation upon interfering with purine metabolism (Fig. 4d, Supplementary Fig. 14, and Supplementary Data 4). Additionally, treatment with MPA also promoted the baseline cytokine release, such as IL-6, IL-1α, and IL-1β, in PBMCs isolated from healthy donors (Supplementary Fig. 15 and Supplementary Data 4).

Given the effects of metabolic manipulation on cytokine release, we next wondered whether administration of exogenous cytokines could reciprocally cause the metabolic alterations. To this end, we treated the PBMCs from healthy donors with a cytokine cocktail (i.e., IL-6, IL-1α, IL-1β, IFN-γ, and TNF-α) for 24 h and collected the cell pellets and culture media for subsequent metabolomics analyses. Our data showed that treatment with exogenous cytokine mixture did not significantly alter the metabolic profile of PBMCs (Supplementary Fig. 16 and Supplementary Data 3). However, cytokine mixture indeed induced a markedly increased release of kynurenine and decreased release of tryptophan to the PBMCs culture media. Several metabolites linked to arginine metabolism, including arginine and glutamate, also showed significantly elevated release from PBMCs (Supplementary Fig. 17 and Supplementary Data 3). These alterations in metabolite levels were consistent with metabolic changes in the serum of COVID-19 patients, suggesting that these metabolic reprogramming may arise, at least in part, from SARS-CoV-2 infection-induced excessive inflammatory cytokines.

Taken together, these data indicate a potential regulatory crosstalk between metabolites and cytokines targeting dysregulated host metabolism may serve as a viable approach to suppress SARS-CoV-2-induced inflammatory cytokine release.

## Discussion

CRS reportedly contributes to vascular damage, immunopathology, and adverse clinical outcomes in COVID-19 patients^2,4,7^. Hence, strategies to constrain the proinflammatory cytokine release are emerging as potential therapies for COVID-19^11,48^. Increasing studies have trialed strategies, including monoclonal antibodies targeting inflammatory cytokines or small-molecule inhibiting the upstream or downstream regulatory pathways, to dampen the inflammatory responses^11,48^. However, better understanding of the driving causes of cytokine storm and identifying potential multi-cytokine blockers are still of urgent need. Considering the previously reported high correlation between metabolism and immune response^20,36,49,50^, and the key role of metabolism in regulating cytokine release upon viral infection^14,29^, we speculated that the metabolism and CRS may correlate well and intervening the dysregulated host metabolism may modulate the SARS-CoV-2-induced CRS. We thus characterized the metabolic and immune profiling in the same COVID-19 patient cohort, and our network correlation analysis between circulating metabolite and cytokine levels and the functional validation experiments by PBMCs ex vivo revealed the potential regulatory role of arginine metabolism, tryptophan metabolism, and purine metabolism in proinflammatory responses.

Our metabolomics data revealed alterations in circulating metabolite levels in patient’s serum samples and identified dysregulated metabolic pathways upon SARS-CoV-2 infection. Consistent with recent studies^24–26^, the metabolites that are associated with arginine metabolism, tryptophan metabolism, TCA cycle, as well as purine and pyrimidine metabolism changed markedly. In our study, we found that metabolites involved in TCA cycle, including citrate, isocitrate, oxalosuccinate, and malate were decreased in COVID-19 patients, which was in line with a recent study demonstrating that metabolites constituting the TCA cycle are generally reduced^25^, potentially suggesting the reduced energy production upon SARS-CoV-2 infection. Importantly, we found succinate was increased, which may enhance the cytokine production through its immunoregulation role as an inflammatory signal^14^. It has also been shown that succinate oxidation is crucial for SARS-CoV-2 replication^29^. The significant reduction in several amino acids, such as tryptophan, glutamine, citrulline, and urea observed in our study, was highly consistent with other studies^24–26,51^. Particularly, the decrease in tryptophan and increase in kynurenine reflecting the hyperactivation of rate-limiting enzyme IDO1 showed the consistency with the independent cohorts from other studies^26,27^. Notably, while we demonstrated that the metabolic reprogramming induced by SARS-CoV-2 infection was radically different from the non-COVID-19 acute upper respiratory tract infection patients, we could not exclude the possibility that other types of pneumonia and/or coronaviruses may result in similar metabolic disturbances as with COVID-19.

The cytokine profiling of COVID-19 patients in our study provided further evidence that the CRS-associated cytokines, such as IL-6, IL-1β, IL-10, IL-18, and IFN-γ, were dramatically elevated in severe patients. Conversely, the increased inflammatory responses experienced progressive reduction accompanied with clinical recovery in mild COVID-19 patients during hospitalization. These findings highlight the need for novel therapies to block multiple cytokines linked to CRS. Emerging evidence suggests the key role of metabolic regulation in cytokine release^20,22,31^. For example, choline uptake and metabolism modulate the IL-1β and IL-18 production in stimulated macrophages^52^. α-ketoglutarate-supplemented diet reportedly induces IL-10 production, thus leading to suppression of chronic inflammation and extension of life span^53^. Particularly, elevated glucose levels in COVID-19 patients promote SARS-CoV-2 replication and cytokine production in monocytes^29^. A very recent study suggests that the high kynurenic acid-to-kynurenine ratio is linked to immune responses and clinical outcomes in male COVID-19 patients^27^. In addition, Vitamin D deficiency associates with the uncontrolled cytokine production and disease severity of COVID-19^54,55^, emphasizing the need of Vitamin D supplementation for COVID-19 treatment. These findings indicate the possibility that modulating dysregulated metabolism may serve as a potential therapeutic approach for controlling cytokine release.

Interestingly, the correlation network analysis in our study revealed that metabolite–cytokine correlations in severe patients were intensified when compared to those in mild patients. In particular, circulating inflammatory cytokine levels, such as IL-6, IP-10, and M-CSF, were highly correlated with metabolites constituting arginine metabolism, tryptophan metabolism, and nucleic acid metabolism in severe patients. Moreover, our time-series clustering analysis in mild patients identified four distinct clusters of longitudinal trajectories delineating the crosstalk between metabolism and inflammatory response. These results suggest that perturbation of metabolic pathways may partially contribute to the consequential CRS in COVID-19. It has been shown that the serum metabolites involved in tryptophan and kynurenine metabolism correlate with IL-6 in COVID-19 patients^26^. Recent studies further identify the important role of IDO1-kynurenine/kynurenic acid-arylhydrocarbon receptors (AhRs) signaling in inflammation and multiple organ injuries in SARS-CoV-2 infection^27,56^, which is consistent with our findings that the increased ratio between circulating kynurenine and tryptophan was positively correlated with proinflammatory cytokines. In addition, our functional validation study also showed that manipulation of tryptophan metabolism by IDO1 inhibitor led to marked decline in proinflammatory cytokines, indicating its therapeutic potential in controlling CRS in the SARS-CoV-2 infection. Arginine is a conditionally essential amino acid for adult mammals and is involved in immune dysfunctions during viral infection^22,31^. Intriguingly, we observed that circulating levels of arginine had a significant positive correlation with CRS-related proinflammatory cytokines. Given that arginine and its downstream metabolites (e.g., ornithine and citrulline) are known to be essential for T cell activation, and thus regulate innate and adaptive immunity^31,57,58^, we speculated that arginine metabolism upon SARS-CoV-2 infection may involve a negative-feedback loop to restrict inflammation. As expected, supplementation of arginine markedly inhibited the elevation in proinflammatory cytokines from PBMCs upon SARS-CoV-2 infection, confirming the anti-inflammatory effect of arginine. We also showed that inhibition of purine metabolism exacerbated inflammatory response. However, the inhibition of pyrimidine biosynthesis pathway reportedly arrests SARS-CoV-2 replication and suppresses inflammatory cytokine release^59^. These results suggest the importance of the balance between purine and pyrimidine metabolism in viral replication and immune response. Notably, treatment with exogenous cytokine cocktail contributed to enhanced release of arginine and kynurenine, and reduced release of tryptophan to culture medium by PBMCs derived from healthy donors, which could be an explanation for the metabolic alterations in the serum of COVID-19 patients. These findings provided clues for the reciprocal regulatory circuits between metabolic alterations and cytokine release, and supported the metabolic intervention as a potential strategy to suppress excessive inflammation. Indeed, combined agents targeting multiple pathogenic factors involved in the hyperinflammation are emerging as the way forward for supportive care for COVID-19^48^. It is therefore possible that cocktails of drugs targeting multiple metabolic pathways for global cytokine blockade might constitute a new class of therapeutic strategy.

Due to the ethical consideration and restricted accessibility to COVID-19 patient samples, our study validated the effects of supplemented metabolites or pharmacological inhibitors in regulating the CRS induced by SARS-CoV-2 infection using the ex vivo model of PBMCs isolated from infected rhesus macaques or healthy donors. Although the isolated PBMCs ex vivo models have been extensively used for the evaluation of cytokine release^23,60^ and rhesus macaque has been reported to be a great animal model recapitulating rapid virus replication and inflammatory responses observed in human upon SARS-CoV-2 infection^45,46^, it would have been ideal to perform such analyses in rhesus macaque in vivo models or PBMCs collected from COVID-19 patients. We tested the impacts of metabolism intervening on the immunological responses in the heterogenous PBMCs. Yet, given that SARS-CoV-2 infection reduces innate antiviral defenses while activates inflammatory cytokine release^61–63^, analyses on sorted subpopulations of immune cells would help to more precisely understand the roles of metabolism in regulating the release of specific cytokines with discriminating functions, proinflammatory or suppressive, from different immune cell types.

In summary, our study performed the metabolic and immune profiling in COVID-19 patients and showed that reprogrammed host metabolism was tightly linked to the burst of proinflammatory cytokines. Beyond providing a resource of metabolism and immunology data to support further investigation of COVID-19, our study also uncovered new insights related to tight correlation between metabolism and cytokine release, and thereby provided a potential therapeutic strategy for the treatment of fatal CRS induced by SARS-CoV-2 infection.

## Methods

### Patients and samples

A total of 44 COVID-19 patients, 20 non-COVD-19 acute upper respiratory tract infection patients, and 17 healthy controls were enrolled in this study. Cross-sectional serum samples from 37 COVID-19 patients were collected from Chongqing Three Gorges Central Hospital, Chongqing Public Health Medical Center and Yongchuan Hospital Affiliated to Chongqing Medical University. Sequential serum samples from 7 mild patients were collected from Yongchuan Hospital Affiliated to Chongqing Medical University with 3-day intervals. Non-COVID-19 acute upper respiratory tract infection patients with respiratory symptoms of common cold, such as fever, cough, nasal congestion, and sore throat, were enrolled from the First and the Second Affiliated Hospitals of Chongqing Medical University. Serum samples from 17 healthy controls were collected from the Second and the Third Affiliated Hospitals of Chongqing Medical University. Patients were confirmed to be infected with SARS-CoV-2 by RT-PCR assays (DAAN Gene) on nasal and pharyngeal swab specimens. Briefly, two target genes, including open reading frame1ab (*ORF1ab*) and nucleocapsid protein (*N*), were simultaneously amplified and tested during RT-PCR (primers can be found in Supplementary Table 2). Primers of RT-PCR testing for SARS-CoV-2 were designed according to the recommendation by the Chinese CDC. PCR cycling: 50 °C for 15 min, 95 °C for 15 min, 45 cycles at 94 °C for 15 s, 55 °C for 45 s (fluorescence collection). Ct values <37 and >40 were defined as positive and negative, respectively, for both genes.

### Clinical data collection

Epidemiologic, demographic, clinical presentations, laboratory tests, treatment, and outcome data were collected from inpatient medical records without allowing for identification. Laboratory data collected for each patient included complete blood count, coagulation profile, serum biochemical tests (including renal and liver function, electrolytes, lactate dehydrogenase, and creatine kinase), serum ferritin, and biomarkers of infection.

### Clinical definitions

Clinical classification was defined based on the COVID-19 Treatment Guidelines (National Health Commission of the People’s Republic of China). A confirmed case of SARS-CoV-2 infection was defined as an individual with nasopharyngeal swabs positive for SARS-CoV-2 nucleic acid by RT-PCR as described above. Severe COVID-19 cases were those meeting any of the following criteria: (1) respiratory distress (≥30 times/min), (2) oxygen saturation ≤ 93% at rest, (3) the arterial partial pressure of oxygen (PaO_2_)/the fraction of inspired oxygen (FiO_2_) ≤300 mmHg. Mild patients were defined as COVID-19 patients with symptoms but could not be classified as severe. Symptoms onset date was defined as the date on which symptoms were first observed. Symptoms included fever, fatigue, dry cough, inappetence, myalgia, dyspnea, expectoration, sore throat, diarrhea, nausea, dizziness, headache, abdominal pain, chill, rhinorrhea, chest stuffiness, or nasal congestion.

### Detection of IgG and IgM against SARS-CoV-2

All serum samples of COVID-19 patients were inactivated at 56 °C for 30 min and stored at −20 °C before testing. IgG and IgM against SARS-CoV-2 in plasma samples were tested using magnetic chemiluminescence enzyme immunoassay kits supplied by Bioscience Co. (approved by the China National Medical Products Administration; approval numbers 20203400183 (IgG) and 20203400182 (IgM)), according to the manufacturer’s instructions. Antibody levels are presented as the measured chemiluminescence values divided by the cutoff (S/CO). Alkaline phosphatase-conjugated Affinipure Goat Anti-Human IgG (Proteintech) was used.

### Cytokine measurement in serum samples from patients and healthy controls

Concentrations of 48 cytokines and chemokines in serum samples were measured using the Bio-Plex Human Cytokine Screening Panel (48-Plex no. 12007283, Bio-Rad) on a Luminex 200 (Luminex Multiplexing Instrument, Merck Millipore) following the manufacturer’s instructions.

### Virus preparation

Viral stocks of SARS-CoV-2 were obtained from the Center of Diseases Control, Guangdong Province, China. Virus samples were amplified on Vero E6 cells and concentrated by ultrafilter system via 300 kDa module (Millipore). Vero E6 cells were cultured in Roswell Park Memorial Institute (RPMI) 1640 medium supplemented with 10% fetal bovine serum (FBS).

### Animals and experimental procedures

For SARS-CoV-2 virus infections, we inoculated a rhesus macaque with total 5 mL of 10^6^ pfu/ mL SARS-CoV-2 intratracheally (2.5 mL) and intranasally (2.5 mL). Body weight, body temperature, and clinical signs were monitored daily. We collected nasal, throat, rectal swabs, and whole blood for viral genome quantification. Real-time RT-PCR was used to quantify viral genome in samples using TaqMan Fast Virus One-step Master Mix (ThermoFisher, USA) and purified viral RNA of SARS-CoV-2 as a standard curve, which was performed on CFX384 Touch Real-Time PCR Detection System (Biorad, USA). Primers and probes of the genome were synthesized according to sequences reported by China CDC (primers can be found in Supplementary Table 2). PCR cycling: 25 °C for 2 min, 50 °C for 15 min, 95 °C for 2 min, then 40 cycles at 95 °C for 5 s and 60 °C for 31 s. For paraffin-embedded sections, tissues were collected and fixed in 10% neutral-buffered formalin, embedded in paraffin, and 5 μM sections were prepared for hematoxylin and eosin (H&E) staining.

### Cytokine measurement in serum samples from rhesus macaque

MILLIPLEX MAP Non-Human Primate Cytokine Magnetic Bead Panel-Immunology Multiplex Assay (PRCYTOMAG-40K, Millipore, USA) was used according to the manufacturer’s protocol, which was performed on Bio-Plex machine. Inflammatory cytokines in this panel included IL-1β, IL-4, IL-5, IL-6, IL-8/CXCL8, G-CSF, GM-CSF, IFN-γ, IL-1RA, IL-2, IL-10, IL-12 p40, IL-13, IL-15, IL-17A/CTLA8, MCP-1/CCL2, MIP-1β/CCL4, MIP-1α/CCL3, sCD40L, TGF-α, TNF-α, VEGF, and IL-18.

### Isolation of PBMCs from peripheral blood

PBMCs were isolated from peripheral blood of mock-infected and SARS-CoV-2-infected rhesus macaques or healthy donors using Ficoll-PaqueTM (Sigma-Aldrich). Peripheral blood sample (4 mL) was drawn into vacutainer tubes. The Ficoll density gradient centrifugation method was used to separate the PBMCs. We diluted the blood with 1× phosphate-buffered saline (PBS) 1:1, and then transferred it to the Ficoll tube. After centrifugation at 1000*g* for 20 min, the buffy coat of PBMCs was pooled and transferred into a 15 mL falcon. PBMCs were then washed twice with 10 mL PBS and centrifuged at 250*g* for 10 min. The cell pellets were resuspended in RPMI 1640 medium.

### Measurement of cytokine release by PBMCs

PBMCs (1 × 10^5^ cells/well) isolated from mock-infected and SARS-CoV-2-infected rhesus macaque or healthy donors were seeded in 96-well plates and treated with different compounds. The compounds including L-arginine (S5634), Epacadostat (S7910), Mycophenolic acid (S2487) were purchased from Selleck. For cytokine measurement of PBMCs derived from rhesus macaques, we used MILLIPLEX MAP Non-Human Primate Cytokine Magnetic Bead Panel-Immunology Multiplex Assay (PRCYTOMAG-40K, Millipore, USA) as mentioned in the section “Cytokine measurement in serum samples from rhesus macaque”. For cytokine measurement of PBMCs derived from healthy donors, we used Bio-Plex Human Cytokine Screening Panel (48-Plex no. 12007283, Bio-Rad) as mentioned in the section “Cytokine measurement in serum samples from patients and healthy controls”.

### Ethical approval

The study was approved by the Ethics Commission of Chongqing Medical University (ref. no. 2020003). Written informed consent was waived by the Ethics Commission of the designated hospitals for emerging infectious diseases. All animal experiments were performed according to protocols approved by the Institutional Animal Care and Use Committee of Institute of Medical Biology, Chinese Academy of Medical Science (Ethics number: DWSP202002 001), and performed in the ABSL-4 facility of Kunming National High-level Biosafety Primate Research Center, Yunnan, China.

### Serum sample preparation for metabolomics

Human serum samples, 20 μL each, were heated at 56 °C for 30 min followed by adding 60 μL ethanol to inactive SARS-CoV-2 virus. The suspension was evaporated to dryness using a SpeedVac concentrator (Thermo Scientific). Metabolites from the serum pellet were extracted with 540 μL 80% methanol in water, followed by vigorous vortex and cooled centrifugation at 4 °C. Then, equal aliquots of the culture medium from each sample (20 μL) were pooled together to make the quality control (QC) samples. The remaining culture medium was divided into two fractions, one for targeted metabolomics and the other for untargeted metabolomics analysis. All the samples were evaporated to dryness.

### PBMCs and cell culture medium sample preparation for metabolomics

The PBMCs isolated from healthy donors were seeded (1 × 10^5^ cells/well) in 24-well plates and treated with different concentrations of cytokines mixture (IL-6, IL-1α, IL-1β, IFN-γ, TNF-α, SinoBiological). After 24 h, 100 μL cell culture medium from each well was pipetted and exacted by adding 400 μL methanol, followed by vigorous vortex and centrifugation. The supernatant was evaporated to dryness. After removing the culture medium and washing with 0.9% NaCl, cell metabolites were extracted with 80% MeOH (methanol:water = 80:20, 500 μL/10^5^ cells), followed by vigorous vortex and centrifugation. The supernatant was evaporated to dryness.

### Targeted metabolomics

For targeted metabolomics, dried metabolites were reconstituted in LC-MS grade water with 0.03% formic acid, vortex-mixed, and centrifuged at 4 °C for 15 min to remove debris. Samples were randomized and blinded before analyzing by LC-MS/MS.

Chromatographic separation was performed on a Nexera UHPLC system (Shimadzu), with a RP-UPLC column (HSS T3, 2.1 mm × 150 mm, 1.8 μm, Waters) and the following gradient: 0–3 min 99% A; 3–15 min 99–1% A; 15–17 min 1% A; 17–17.1 min 1–99% A; 17.1–20 min 99% A. Mobile phase A was 0.03% formic acid in water. Mobile phase B was 0.03% formic acid in acetonitrile. The flow rate was 0.25 mL/min, the column was at 35 °C and the autosampler was at 4 °C. Mass data acquisition was performed using an AB QTRAP 6500+ triple quadrupole mass spectrometer (SCIEX, Framingham, MA) in multiple reaction monitoring (MRM) mode for the detection of 258 unique endogenous water-soluble metabolites as previously described, with some modifications^22,64^. Chromatogram review and peak area integration were performed using MultiQuant 3.0.2 (SCIEX, Framingham, MA).

### Untargeted metabolomics

For untargeted metabolomics, dried samples were reconstituted in acetonitrile/water mixture (v/v, 1:1). After vortex, samples were centrifuged at 4 °C for 15 min to remove debris, samples were randomized and blinded before LC-MS/MS analysis.

Chromatographic separation was performed on an Agilent 1290 infinity II LC system, with an Agilent Eclipse Plus C18 column (2.1 mm × 100 mm, 1.8 μm). The gradient was set as follows: 0–2 min 95% A; 2–20 min 95-0% A; 20–25 min 0% A; post-run time for equilibration, 5 min in 95% A. Water containing 0.1% formic acid and acetonitrile containing 0.1% formic acid acted as mobile phase A and B in the positive-ion mode of mass spectrometry analysis. While in the negative-ion mode of mass spectrometry analysis, the 0.1% formic acid was replaced with 1 mM ammonium fluoride. The flow rate was set as 0.3 mL/min and the temperatures of the column and autosampler were set as 40 and 4 °C, respectively. The data acquisition was performed on a 6546 Q-TOF mass spectrometry equipped with a dual electrospray (ESI) ion source (Agilent Technologies, Santa Clara, CA). The optimized ESI Q-TOF parameters were set as follows: the temperature and flow rate of sheath gas were 350 °C and 11 L/min; the voltages of capillary, fragmentor, and skimmer were set as 4000, 140, and 65 V, respectively. The spectra were internally mass calibrated in real time by continuous infusion of a reference mass solution using an isocratic pump connected to a dual sprayer feeding into an ESI source. MassHunter Acquisition software (Agilent Technologies, Santa Clara, CA, v10.1) was employed to perform data acquisition.

Raw data were obtained from the Agilent Masshunter Workstation (Profinder software, v10.0) and the metabolite features were extracted by excluding missing values based on 80% rule^65^. The metabolites were identified based on their retention times, accurate masses, MS/MS spectra, and isotopic patterns. Further, to expand the qualitative coverage of specific metabolic pathways, the Agilent Pathway to PCDL software (vB.08.00) was employed to perform targeted extraction of metabolites from the raw data. According to the above criteria, a total of 155 metabolites were eventually identified.

### Normalization and integration of targeted and untargeted metabolomics data

For both targeted and untargeted metabolomics, QC samples composed of an equal aliquot of all test samples were prepared and inserted in an interval of ten test samples to monitor the stability of instrument and normalize the variations during the run. This served as an additional QC measure of analytical performance and a reference for normalizing raw metabolomics data across samples.

To remove potential inter-batch variations, the mean peak area of each metabolite from all the QC samples in all given batches (QC_all_), as well as the mean peak area of each metabolite from the QC samples that are the most adjacent to a given group of test samples (QC_adj_) were first calculated. The ratio between these two mean peak areas for each metabolite was computed by dividing the same QC_all_ by each QC_adj_ and used as the normalization factor for each given group of test samples. The peak area of each metabolite from each test sample was normalized by multiplying their corresponding normalization ratio to obtain the normalized peak areas. In addition, to effectively correct the sample-to-sample variation in biomass that may contribute to systematic differences in metabolite abundance detected by LC-MS, we generated the scaled data by comparing the normalized peak area of each metabolite to the sum of the normalized peak area from all the detected (for targeted metabolomics) or identified metabolites (for untargeted metabolomics) in that given sample.

Our validation analyses suggested that these normalization and scaling steps could effectively correct both the inter-sample artificial differences in sample biomass and inter-batch systematic variations in detected metabolite abundance.

A total of 36 metabolites overlapped among the 134 metabolites detected by the targeted metabolomics analysis and 155 metabolites detected by the untargeted metabolomics analysis. For the overlap metabolites identified from targeted and untargeted metabolomics, data from targeted method were used for the subsequent analysis. Finally, 253 metabolites were integrated into the final metabolomics data matrix including 134 metabolites from targeted metabolic profiling and 119 metabolites from untargeted metabolic profiling. The final metabolomics data matrix (102 rows of patients/controls and 253 columns of metabolites) used for the downstream analyses or modeling is included in Supplementary Data 3.

### Partial least squares discrimination analysis (PLS-DA)

PLS-DA was performed on normalized metabolomics data using SIMCA-P software (v14.1, Umetrics, Umea, Sweden) and unit variance (UV) scaling was utilized before multivariate analysis.

### *t*-Distributed stochastic neighbor embedding (*t*-SNE) dimensionality reduction

*t*-SNE scatterplots were generated of log10-transformed metabolomics data using R Rtsne (v0.15) package with perplexity of 5 and theta of 0.01.

### Identification of significantly altered metabolites and cytokines in mild and severe COVID-19 patients

Mann-Whitney *U* test followed by Benjamini-Hochberg (BH) multiple comparisons test was performed by R stat (v3.6.0) package. Serum cytokines with FDR < 0.05 and metabolites with FDR < 0.05, fold change >1.25 or <0.8 were considered significant and used for subsequent analysis.

### Longitudinal trajectory analysis of serum metabolites and cytokines

To estimate serum cytokine and metabolite longitudinal trajectories of follow-up mild COVID-19 patients, generalized additive model (GAM) adjusted for age and gender was fitted for each cytokine and metabolite. GAM was performed by R mgcv (v1.8-31) package with default parameters.

Mann-Whitney *U* test and BH multiple comparison test were carried out at each time-point to identify significant altered cytokines and metabolites compared with healthy controls (base lines). Cytokines and metabolites with FDR < 0.05 were considered significant. Longitudinal clustering was performed using the *z*-scaled abundance of significant cytokines and metabolites by R Mfuzz (v2.44.0) package, and parameter *m* was set to 1.5.

### Correlation analysis of serum metabolites and cytokines

Correlations between cytokines and metabolites were analyzed by linear regressions models after adjusting for gender and age. Linear regression models conducted by R lm base function were calculated using Log10-transformed abundance of metabolites and cytokines in mild patients, severe patients, and follow-up patients.

1 $${\mathrm{Log}}_{10}\left( {{\mathrm{Cytokine}}\,{\mathrm{abundance}}} \right) = {\mathrm{Gender}} + {\mathrm{Age}} + {\mathrm{Log}}_{10}\left( {{\mathrm{Metabolite}}\,{\mathrm{abundance}}} \right)$$

*P* values were corrected using BH multiple comparisons test. Correlations with FDR < 0.1 were considered significant and used for subsequent analysis.

Then, based on the significant cytokine–metabolite correlations in severe patients, weighted, undirected correlation networks were built by R igraph (v1.2.5) package. Clusters were determined based on fast greedy modularity optimization algorithm.

### KEGG pathway analysis

KEGG metabolic pathways and involved metabolites were downloaded from KEGG API (https://www.kegg.jp/kegg/rest/keggapi.html). Significant enriched KEGG pathways were determined by R clusterProfiler (v3.12.0) package with BH multiple comparison test as FDR < 0.1 and enriched for at least 3 metabolites (for significantly altered metabolites), FDR < 0.1 and enriched for at least 3 cytokines (for cytokine-associated metabolites), or FDR < 0.05 (for metabolites in each trajectory cluster in follow-up patients).

For metabolites with progressive change in mild and severe patients, metabolites with abundance in severe patients > mild patients > healthy controls or severe patients < mild patients < healthy controls, and showed significance in severe patients compared with mild patients were considered consistently altered. Based on these metabolites, metabolic pathways with FDR < 0.1 were considered significantly enriched.

### Statistical analysis

Quantification methods and statistical analysis methods for metabolite and cytokine analyses were mainly described and referenced in the respective subsections in Methods.

Additionally, statistical significance of cytokines in cell culture media and relative abundance of metabolites in PBMC pellets or culture media between different conditions were considered using one-way ANOVA followed by BH multiple comparison test, which were performed by GraphPad prism (v8.2.1).

### Reporting summary

Further information on research design is available in the Nature Research Reporting Summary linked to this article.

## Supplementary information

Supplementary Information

Reporting Summary

Description of Additional Supplementary Files

Supplementary Data 1

Supplementary Data 2

Supplementary Data 3

Supplementary Data 4

## Data Availability

Clinical information of COVID-19 patients, non-COVID-19 acute upper respiratory tract infection patients and healthy controls are included in Supplementary Data 1. Raw metabolomics mass spectrometry data are included in Supplementary Data 2. Normalized metabolomics data are included in Supplementary Data 3. Cytokine and chemokine abundances are included in Supplementary Data 4. Source data are provided with this paper.

## Source data

Source Data

## Code availability

Codes for processing metabolomics, cytokine data and correlation analysis are available at https://github.com/amberxn/COVID19_NCOMMS.

## References

[1] Huang C (2020). Clinical features of patients infected with 2019 novel coronavirus in Wuhan, China. Lancet.

[2] Mehta P (2020). COVID-19: consider cytokine storm syndromes and immunosuppression. Lancet.

[3] Cao X (2020). COVID-19: immunopathology and its implications for therapy. Nat. Rev. Immunol..

[4] Mangalmurti N, Hunter CA (2020). Cytokine storms: understanding COVID-19. Immunity.

[5] Zhang X (2020). Viral and host factors related to the clinical outcome of COVID-19. Nature.

[6] Lucas C (2020). Longitudinal analyses reveal immunological misfiring in severe COVID-19. Nature.

[7] Del Valle DM (2020). An inflammatory cytokine signature predicts COVID-19 severity and survival.. Nat. Med..

[8] Liang AG (2020). A dynamic COVID-19 immune signature includes associations with poor prognosis.. Nat. Med..

[9] Strohbehn GW (2021). COVIDOSE: A Phase II Clinical Trial of Low-Dose Tocilizumab in the Treatment of Noncritical COVID-19 Pneumonia. Clin. Pharmacol. Ther..

[10] Walz L (2021). JAK-inhibitor and type I interferon ability to produce favorable clinical outcomes in COVID-19 patients: a systematic review and meta-analysis. BMC Infect. Dis..

[11] Luo W (2020). Targeting JAK-STAT signaling to control cytokine release syndrome in COVID-19. Trends Pharmacol. Sci..

[12] Oren R, Farnham AE, Saito K, Milofsky E, Karnovsky ML (1963). Metabolic patterns in three types of phagocytizing cells. J. Cell Biol..

[13] O’Neill LA, Kishton RJ, Rathmell J (2016). A guide to immunometabolism for immunologists. Nat. Rev. Immunol..

[14] Tannahill GM (2013). Succinate is an inflammatory signal that induces IL-1beta through HIF-1alpha. Nature.

[15] Al-Khalili L (2006). Signaling specificity of interleukin-6 action on glucose and lipid metabolism in skeletal muscle. Mol. Endocrinol..

[16] Qing H (2020). Origin and function of stress-induced IL-6 in murine models. Cell.

[17] Lampropoulou V (2016). Itaconate links inhibition of succinate dehydrogenase with macrophage metabolic remodeling and regulation of inflammation. Cell Metab..

[18] Bambouskova M (2018). Electrophilic properties of itaconate and derivatives regulate the IκBζ-ATF3 inflammatory axis. Nature.

[19] Mills EL (2018). Itaconate is an anti-inflammatory metabolite that activates Nrf2 via alkylation of KEAP1. Nature.

[20] Saez-Cirion A, Sereti I (2021). Immunometabolism and HIV-1 pathogenesis: food for thought. Nat. Rev. Immunol..

[21] Chan KR (2019). Metabolic perturbations and cellular stress underpin susceptibility to symptomatic live-attenuated yellow fever infection. Nat. Med..

[22] Li XK (2018). Arginine deficiency is involved in thrombocytopenia and immunosuppression in severe fever with thrombocytopenia syndrome.. Sci. Transl. Med..

[23] Wang Q (2020). O-GlcNAc transferase promotes influenza A virus-induced cytokine storm by targeting interferon regulatory factor-5. Sci. Adv..

[24] Shen B (2020). Proteomic and metabolomic characterization of COVID-19 patient sera. Cell.

[25] Song JW (2020). Omics-driven systems interrogation of metabolic dysregulation in COVID-19 pathogenesis. Cell Metab..

[26] Thomas T (2020). COVID-19 infection alters kynurenine and fatty acid metabolism, correlating with IL-6 levels and renal status.. JCI Insight.

[27] Cai, Y. et al. Kynurenic acid underlies sex-specific immune responses to COVID-19. Preprint at *medRxiv* 10.1101/2020.09.06.20189159 (2020).

[28] Su Y (2020). Multi-omics resolves a sharp disease-state shift between mild and moderate COVID-19. Cell.

[29] Codo AC (2020). Elevated glucose levels favor SARS-CoV-2 infection and monocyte response through a HIF-1alpha/glycolysis-dependent axis. Cell Metab..

[30] Guan WJ (2020). Clinical characteristics of coronavirus disease 2019 in China. N. Engl. J. Med..

[31] Bronte V, Zanovello P (2005). Regulation of immune responses by L-arginine metabolism. Nat. Rev. Immunol..

[32] Cervenka I, Agudelo LZ, Ruas JL (2017). Kynurenines: tryptophan’s metabolites in exercise, inflammation, and mental health.. Science.

[33] Belladonna ML, Orabona C (2020). Potential benefits of tryptophan metabolism to the efficacy of tocilizumab in COVID-19. Front. Pharmacol..

[34] Mesquita I (2016). Exploring NAD+ metabolism in host–pathogen interactions. Cell Mol. Life Sci..

[35] Heer CD (2020). Coronavirus infection and PARP expression dysregulate the NAD Metabolome: an actionable component of innate immunity. J. Biol. Chem..

[36] Beisel WR (1975). Metabolic response to infection. Annu. Rev. Med..

[37] Wilk AJ (2020). A single-cell atlas of the peripheral immune response in patients with severe COVID-19. Nat. Med..

[38] Long QX (2020). Antibody responses to SARS-CoV-2 in patients with COVID-19. Nat. Med..

[39] Moore JB, June CH (2020). Cytokine release syndrome in severe COVID-19. Science.

[40] Couper KN, Blount DG, Riley EM (2008). IL-10: the master regulator of immunity to infection. J. Immunol..

[41] Chen J (2021). Functional comparison of IFN-alpha subtypes reveals potent HBV suppression by a concerted action of IFN-alpha and -gamma signaling. Hepatology.

[42] Zhou Q (2020). Interferon-alpha2b treatment for COVID-19. Front. Immunol..

[43] Lu S (2020). Comparison of nonhuman primates identified the suitable model for COVID-19. Signal Transduct. Target Ther..

[44] Aid M (2020). Vascular disease and thrombosis in SARS-CoV-2-infected rhesus macaques. Cell.

[45] Chandrashekar A (2020). SARS-CoV-2 infection protects against rechallenge in rhesus macaques. Science.

[46] Munster VJ (2020). Respiratory disease in rhesus macaques inoculated with SARS-CoV-2.. Nature.

[47] Shan C (2020). Infection with novel coronavirus (SARS-CoV-2) causes pneumonia in rhesus macaques. Cell Res..

[48] Harrison C (2020). Focus shifts to antibody cocktails for COVID-19 cytokine storm. Nat. Biotechnol..

[49] Wang A, Luan HH, Medzhitov R (2019). An evolutionary perspective on immunometabolism.. Science.

[50] Sica A (2020). Immunometabolic status of COVID-19 cancer patients. Physiol. Rev..

[51] Bruzzone C (2020). SARS-CoV-2 infection dysregulates the metabolomic and lipidomic profiles of serum. iScience.

[52] Sanchez-Lopez E (2019). Choline uptake and metabolism modulate macrophage IL-1beta and IL-18 production. Cell Metab..

[53] Asadi Shahmirzadi A (2020). Alpha-ketoglutarate, an endogenous metabolite, extends lifespan and compresses morbidity in aging mice. Cell Metab..

[54] Mardani R (2020). Association of vitamin D with the modulation of the disease severity in COVID-19.. Virus Res..

[55] Daneshkhah A (2020). Evidence for possible association of vitamin D status with cytokine storm and unregulated inflammation in COVID-19 patients.. Aging Clin. Exp. Res..

[56] Turski WA, Wnorowski A, Turski GN, Turski CA, Turski L (2020). AhR and IDO1 in pathogenesis of Covid-19 and the “Systemic AhR Activation Syndrome:” Translational review and therapeutic perspectives.. Restor. Neurol. Neurosci..

[57] Chatterjee S (2006). Arginine metabolic pathways determine its therapeutic benefit in experimental heatstroke: role of Th1/Th2 cytokine balance. Nitric Oxide.

[58] Burrack KS, Morrison TE (2014). The role of myeloid cell activation and arginine metabolism in the pathogenesis of virus-induced diseases. Front. Immunol..

[59] Luban J (2021). The DHODH inhibitor PTC299 arrests SARS-CoV-2 replication and suppresses induction of inflammatory cytokines.. Virus Res..

[60] Lee ACY (2017). Avian influenza virus A H7N9 infects multiple mononuclear cell types in peripheral blood and induces dysregulated cytokine responses and apoptosis in infected monocytes. J. Gen. Virol..

[61] Hadjadj J (2020). Impaired type I interferon activity and inflammatory responses in severe COVID-19 patients. Science.

[62] Tay MZ, Poh CM, Renia L, MacAry PA, Ng LFP (2020). The trinity of COVID-19: immunity, inflammation and intervention. Nat. Rev. Immunol..

[63] Blanco-Melo D (2020). Imbalanced host response to SARS-CoV-2 drives development of COVID-19. Cell.

[64] Huang F (2018). Inosine monophosphate dehydrogenase dependence in a subset of small cell lung cancers. Cell Metab..

[65] Bijlsma S (2006). Large-scale human metabolomics studies: a strategy for data (pre-) processing and validation. Anal. Chem..

