# Supplementary material for: Integrated cytokine and metabolite analysis reveals immunometabolic reprogramming in COVID-19 patients with therapeutic implications
Source: Nat Commun. 2021 Mar 12;12:1618. doi: 10.1038/s41467-021-21907-9 (PMC7955129; doi:10.1038/s41467-021-21907-9)
Supplement: Supplementary file 1 — Supplementary Information [file 41467_2021_21907_MOESM1_ESM.pdf]

# Integrated cytokine and metabolite analysis reveals immunometabolic reprogramming in COVID-19 patients with therapeutic implications

Nan Xiao[1,8], Meng Nie[1,2,8], Huanhuan Pang[1,2,8], Bohong Wang[1,2,8], Jieli Hu[3,8], Xiangjun Meng[1], Ke Li[4], Xiaorong Ran[5], Quanxin Long[3], Haijun Deng[3], Na Chen[1], Shao Li[6], Ni Tang[3]*, Ailong Huang[3]*, and Zeping Hu[1,2,7]*

[1]School of Pharmaceutical Sciences, Tsinghua University, Beijing, 100084, China.

[2]Tsinghua-Peking Joint Center for Life Sciences, Tsinghua University, Beijing, 100084, China.

[3]Key Laboratory of Molecular Biology for Infectious Diseases (Ministry of Education), Chongqing Medical University, Chongqing, China.

[4]NHC Key Laboratory of Biotechnology of Antibiotics, Institute of Medicinal Biotechnology, Chinese Academy of Medical Sciences & Peking Union Medical College, Beijing, 100050, China

[5]Agilent Technologies (China), No. 3, Wang Jing Bei Lu, Chaoyang District, Beijing, 100102, China

[6]Institute for TCM-X, MOE Key Laboratory of Bioinformatics / Bioinformatics Division, BNRIST, Department of Automation, Tsinghua University, Beijing 100084, China

[7]Beijing Frontier Research Center for Biological Structure, Tsinghua University, Beijing, 100084, China.

[8]These authors contributed equally

***Correspondence:**

nitang@cqmu.edu.cn (N.T.), ahuang@cqmu.edu.cn (A.H.), zeping_hu@tsinghua.edu.cn (Z.H.)

# Supplementary Figures

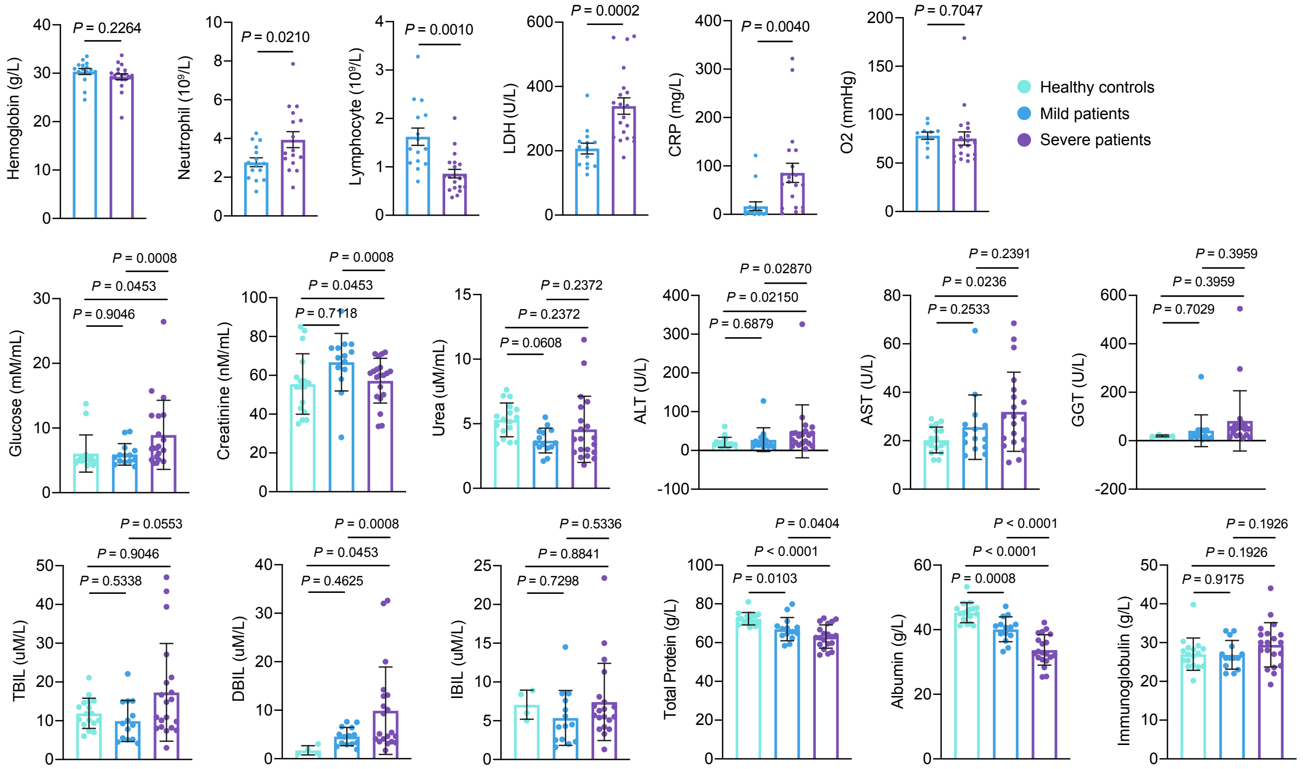


**Supplementary Fig. 1 Clinical information of mild and severe COVID-19 patients.** Clinical information of healthy controls (n = 17), mild (n = 14) and severe (n = 23) COVID-19 patients. Unpaired, two-sided *t* test or one-way ANOVA followed by Benjamini-Hochberg (BH) multiple comparison test. Data are presented as mean ± SEM. with individual data points shown. LDH, Lactic Acid Dehydrogenase. CRP, C-Reactive Protein. ALT, Alanine Aminotransferase. AST, Aspartate Aminotransferase. GGT, Gamma-Glutamyl Transferase. TBIL, Total Bilirubin. DBIL, Direct Bilirubin. IBIL, Indirect Bilirubin.

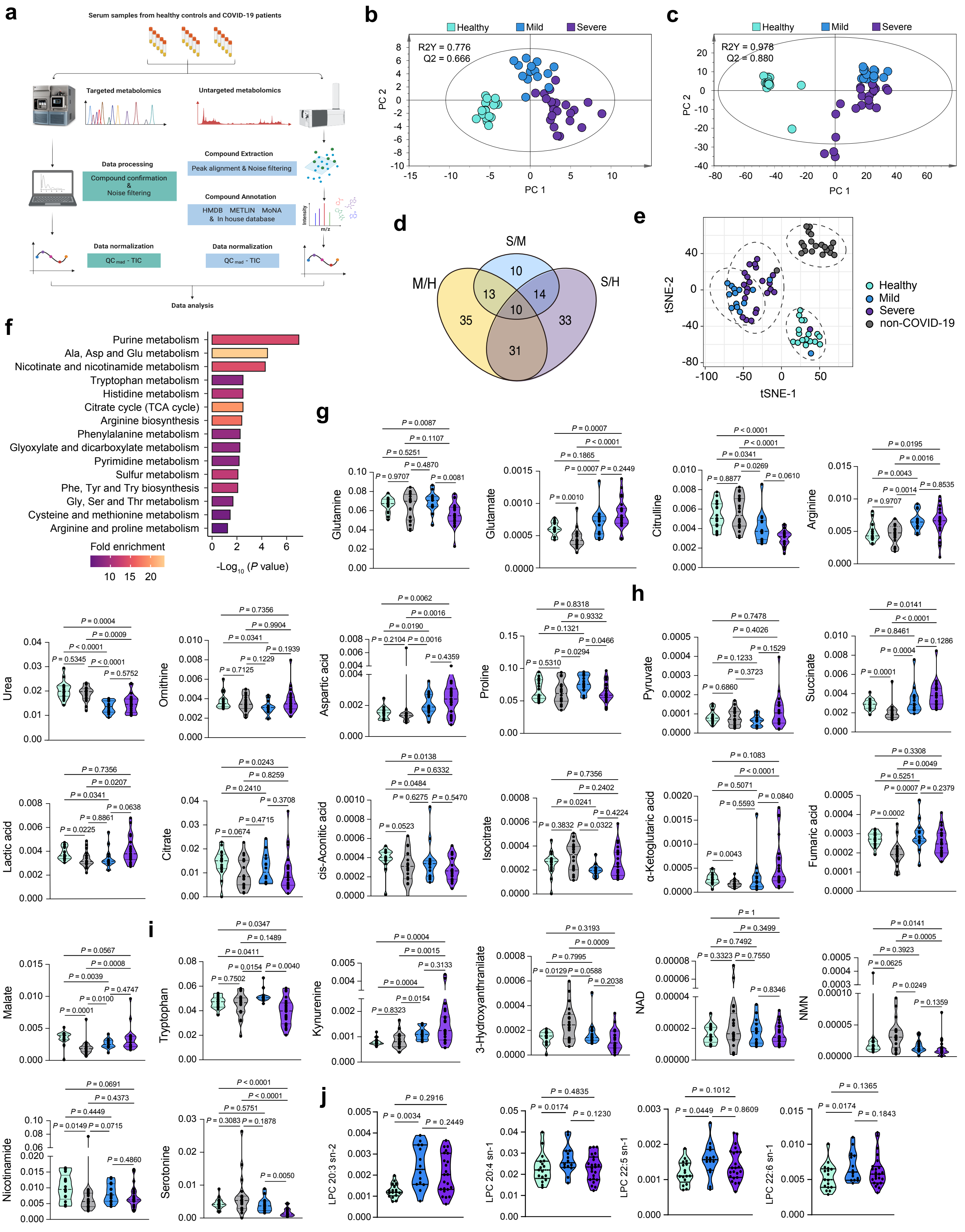
a
Serum samples from healthy controls and COVID-19 patients
Targeted metabolomics
Untargeted metabolomics
Data processing
Compound confirmation & Noise filtering
Compound Extraction
Peak alignment & Noise filtering
Compound Annotation
HMDB METLIN MoNA & In house database
Data normalization
QC mad - TIC
Data normalization
QC mad - TIC
Data analysis
b
Healthy
Mild
Severe
R2Y = 0.776
Q2 = 0.666
PC 1
PC 2
c
Healthy
Mild
Severe
R2Y = 0.978
Q2 = 0.880
PC 1
PC 2
d
S/M
M/H
S/H
10
13
14
10
35
33
31
e
tSNE-1
tSNE-2
Healthy
Mild
Severe
non-COVID-19
f
Purine metabolism
Ala, Asp and Glu metabolism
Nicotinate and nicotinamide metabolism
Tryptophan metabolism
Histidine metabolism
Citrate cycle (TCA cycle)
Arginine biosynthesis
Phenylalanine metabolism
Glyoxylate and dicarboxylate metabolism
Pyrimidine metabolism
Sulfur metabolism
Phe, Tyr and Try biosynthesis
Gly, Ser and Thr metabolism
Cysteine and methionine metabolism
Arginine and proline metabolism
Fold enrichment
10 15 20
-Log10 (P value)
g
Glutamine
Glutamate
Citrulline
Arginine
Urea
Ornithine
Aspartic acid
Proline
h
Pyruvate
Succinate
Lactic acid
Citrate
cis-Aconitic acid
Isocitrate
α-Ketoglutaric acid
Fumaric acid
Malate
i
Tryptophan
Kynurenine
3-Hydroxyanthranilate
NAD
NMN
Nicotinamide
Serotonine
j
LPC 20:3 sn-2
LPC 20:4 sn-1
LPC 22:5 sn-1
LPC 22:6 sn-1

**Supplementary Fig. 2 Overview of metabolomics data in COVID-19 patients. a,** General workflow of metabolomic profiling experiments and data analysis. **b-c,** Partial Least Squares Discriminant Analysis (PLS-DA) of targeted metabolomics data **(b)** and untargeted metabolomics data **(c)** of healthy controls, mild and severe COVID-19 patients. **d,** Venn diagram depicting the number of significantly altered serum metabolites in each group after integrating targeted and untargeted metabolomics data. **e,** t-SNE plot distributed healthy controls (n = 17), mild patients (n = 14), severe patients (n = 23) and non-COVID-19 patients (n = 20) according to serum metabolites detected from targeted metabolomics. **f,** Metabolic pathways enriched based on metabolites consistently increased or decreased in mild and severe patients compared with healthy controls. Fold enrichment is represented by color intensity. One-sided Fisher's exact test followed by BH multiple comparison test. **g-i,** Relative abundance of metabolites involved in arginine metabolism **(g)**, TCA cycle **(h)**, tryptophan and $NAD^+$ metabolism **(i)**. **j.** Relative abundance of indicated lysophospholipids. Two-sided Mann-Whitney U test followed by BH multiple comparison test.

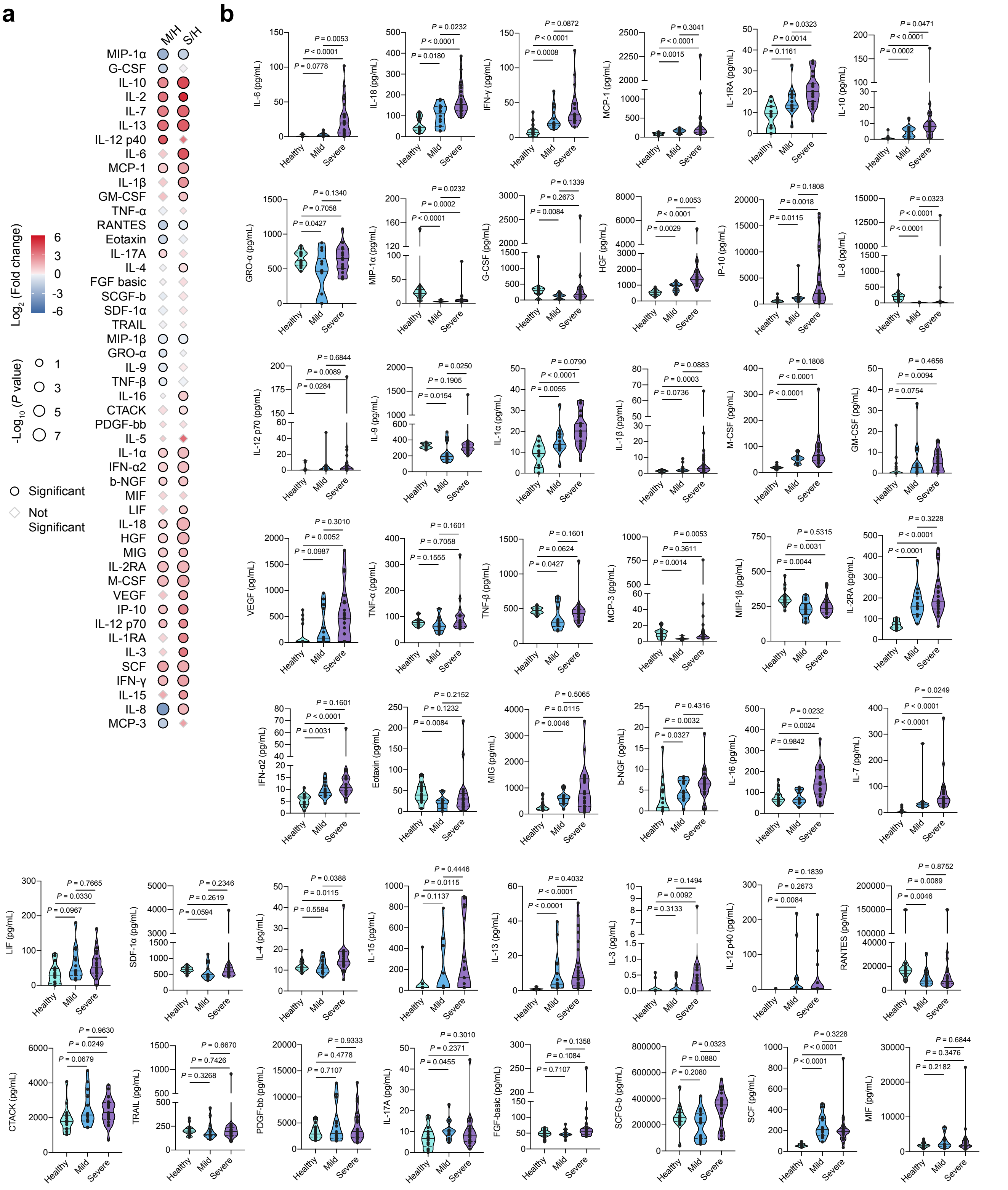


**Supplementary Fig. 3 Overview of cytokine data in COVID-19 patients. a,** Alterations of cytokine abundance in mild (n = 14) or severe (n = 23) patients compared with healthy controls (n = 17). Log2 transformed fold change is represented by color intensity. Shape represents alteration significance, and

FDR is size-coded. Two-sided Mann-Whitney U test followed by BH multiple comparison test with FDR < 0.05. **b,** Violin plots comparing serum cytokines abundance in healthy controls, mild patients and severe patients. Data are presented as mean and quantiles with individual data points shown. Two-sided Mann-Whitney U test followed by BH multiple comparison test.

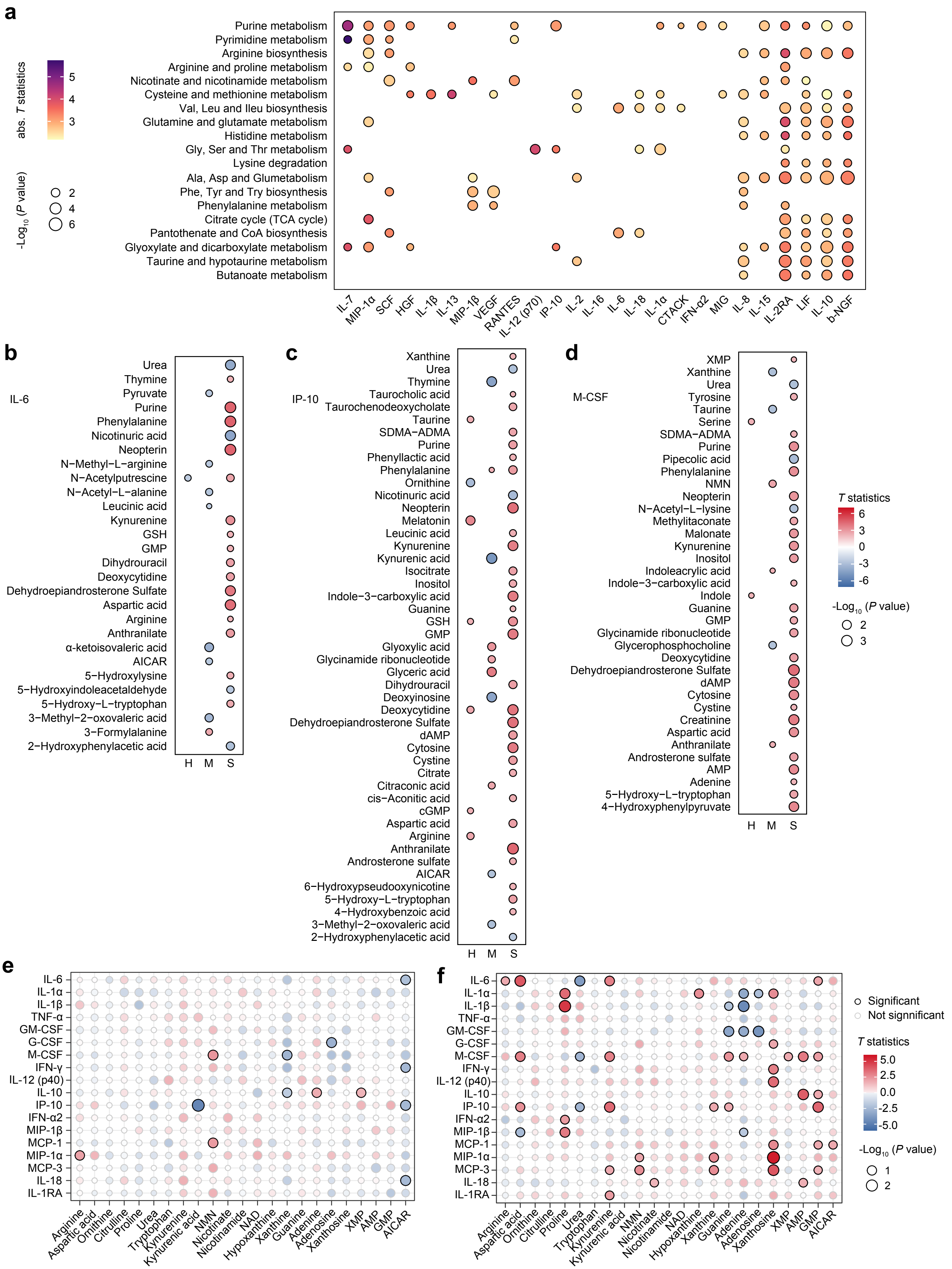
a
abs. T statistics
5
4
3
-Log10 (P value)
2
4
6
Purine metabolism
Pyrimidine metabolism
Arginine biosynthesis
Arginine and proline metabolism
Nicotinate and nicotinamide metabolism
Cysteine and methionine metabolism
Val, Leu and Ileu biosynthesis
Glutamine and glutamate metabolism
Histidine metabolism
Gly, Ser and Thr metabolism
Lysine degradation
Ala, Asp and Glumetabolism
Phe, Tyr and Try biosynthesis
Phenylalanine metabolism
Citrate cycle (TCA cycle)
Pantothenate and CoA biosynthesis
Glyoxylate and dicarboxylate metabolism
Taurine and hypotaurine metabolism
Butanoate metabolism
IL-7
MIP-1α
SCF
HGF
IL-1β
IL-13
MIP-1β
VEGF
RANTES
IL-12 (p70)
IP-10
IL-2
IL-16
IL-6
IL-18
IL-1α
CTACK
IFN-α2
MIG
IL-8
IL-15
IL-2RA
LIF
IL-10
b-NGF
b
IL-6
Urea
Thymine
Pyruvate
Purine
Phenylalanine
Nicotinuric acid
Neopterin
N−Methyl−L−arginine
N−Acetylputrescine
N−Acetyl−L−alanine
Leucinic acid
Kynurenine
GSH
GMP
Dihydrouracil
Deoxycytidine
Dehydroepiandrosterone Sulfate
Aspartic acid
Arginine
Anthranilate
α-ketoisovaleric acid
AICAR
5−Hydroxylysine
5−Hydroxyindoleacetaldehyde
5−Hydroxy−L−tryptophan
3−Methyl−2−oxovaleric acid
3−Formylalanine
2−Hydroxyphenylacetic acid
H
M
S
c
IP-10
Xanthine
Urea
Thymine
Taurocholic acid
Taurochenodeoxycholate
Taurine
SDMA−ADMA
Purine
Phenyllactic acid
Phenylalanine
Ornithine
Nicotinuric acid
Neopterin
Melatonin
Leucinic acid
Kynurenine
Kynurenic acid
Isocitrate
Inositol
Indole−3−carboxylic acid
Guanine
GSH
GMP
Glyoxylic acid
Glycinamide ribonucleotide
Glyceric acid
Dihydrouracil
Deoxyinosine
Deoxycytidine
Dehydroepiandrosterone Sulfate
dAMP
Cytosine
Cystine
Citrate
Citraconic acid
cis−Aconitic acid
cGMP
Aspartic acid
Arginine
Anthranilate
Androsterone sulfate
AICAR
6−Hydroxypseudooxynicotine
5−Hydroxy−L−tryptophan
4−Hydroxybenzoic acid
3−Methyl−2−oxovaleric acid
2−Hydroxyphenylacetic acid
H
M
S
d
M-CSF
XMP
Xanthine
Urea
Tyrosine
Taurine
Serine
SDMA−ADMA
Purine
Pipecolic acid
Phenylalanine
NMN
Neopterin
N−Acetyl−L−lysine
Methylitaconate
Malonate
Kynurenine
Inositol
Indoleacrylic acid
Indole−3−carboxylic acid
Indole
Guanine
GMP
Glycinamide ribonucleotide
Glycerophosphocholine
Deoxycytidine
Dehydroepiandrosterone Sulfate
dAMP
Cytosine
Cystine
Creatinine
Aspartic acid
Anthranilate
Androsterone sulfate
AMP
Adenine
5−Hydroxy−L−tryptophan
4−Hydroxyphenylpyruvate
H
M
S
T statistics
6
3
0
-3
-6
-Log10 (P value)
2
3
e
IL-6
IL-1α
IL-1β
TNF-α
GM-CSF
G-CSF
M-CSF
IFN-γ
IL-12 (p40)
IL-10
IP-10
IFN-α2
MIP-1β
MCP-1
MIP-1α
MCP-3
IL-18
IL-1RA
Arginine
Aspartic acid
Ornithine
Citrulline
Proline
Urea
Tryptophan
Kynurenine
Kynurenic acid
NMN
Nicotinate
Nicotinamide
NAD
Hypoxanthine
Xanthine
Guanine
Adenine
Adenosine
Xanthosine
XMP
AMP
GMP
AICAR
f
IL-6
IL-1α
IL-1β
TNF-α
GM-CSF
G-CSF
M-CSF
IFN-γ
IL-12 (p40)
IL-10
IP-10
IFN-α2
MIP-1β
MCP-1
MIP-1α
MCP-3
IL-18
IL-1RA
Arginine
Aspartic acid
Ornithine
Citrulline
Proline
Urea
Tryptophan
Kynurenine
Kynurenic acid
NMN
Nicotinate
Nicotinamide
NAD
Hypoxanthine
Xanthine
Guanine
Adenine
Adenosine
Xanthosine
XMP
AMP
GMP
AICAR
Significant
Not significant
T statistics
5.0
2.5
0.0
-2.5
-5.0
-Log10 (P value)
1
2

**Supplementary Fig.4 Comparisons of cytokine-metabolite correlations between mild and severe patients. a,** Pathway enrichment analysis of metabolites significantly associated with the indicated cytokines in mild patients (n = 14). Two-sided *t* test followed by BH multiple comparison test with FDR < 0.1. "abs. *T* statistics" is the mean absolute *T* statistics of significantly changed metabolites in the pathway and is represented by color intensity. The dot size represents pathway significance (one-sided Fisher's exact test followed by BH multiple comparison test with FDR < 0.1). **b-d,** Metabolites significantly correlated with IL-6 **(b)**, IP-10 **(c)**, M-CASF **(d)** in healthy controls (n = 17), mild patients (n = 14) and severe patients (n = 23). **e-f,** Correlation of core CRS-related cytokines with metabolites in arginine metabolism, tryptophan and $NAD^+$ metabolism and purine metabolism in mild **(e)** and severe patients **(f)**. Two-sided *t* test followed by BH multiple comparison test with FDR < 0.1. *T* statistics is represented by color intensity, and size represents significance.

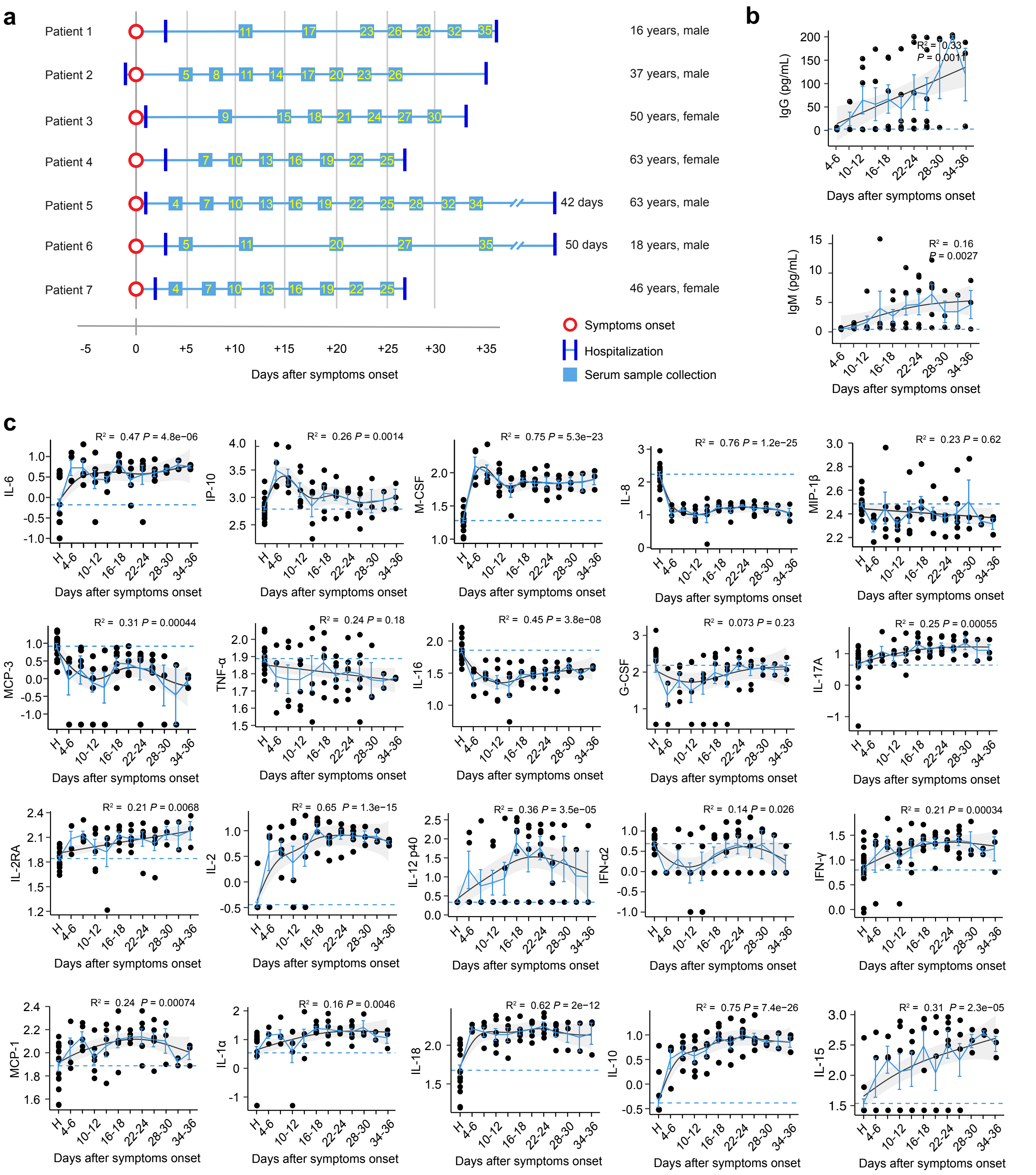


**Supplementary Fig. 5 Longitudinal cytokine trajectories in follow-up mild COVID-19 patients. a,** Schematic diagram of sample collection for follow-up patients (n = 7). Blue squares indicate the time of serum collection; red circles represent the time of symptoms onset; dark blue lines represent the time of hospitalization. **b-c,** Longitudinal abundance of antibodies **(b)**, Log10 transformed abundance of cytokine

and chemokine **(c)** trajectories of follow-up patients (n = 7) in an interval of 3 days. Blue solid lines pass through the mean of each measurement at the specific time interval, and dotted lines represent the mean of measurements in healthy controls (n = 17). Generalized addictive model (GAM) regression lines are indicated by the black solid lines, with 95% confidence intervals for the regression lines donated by gray filled areas. *P* value was assessed by one-way ANOVA. Data are presented as mean ± SEM. with individual data points shown.

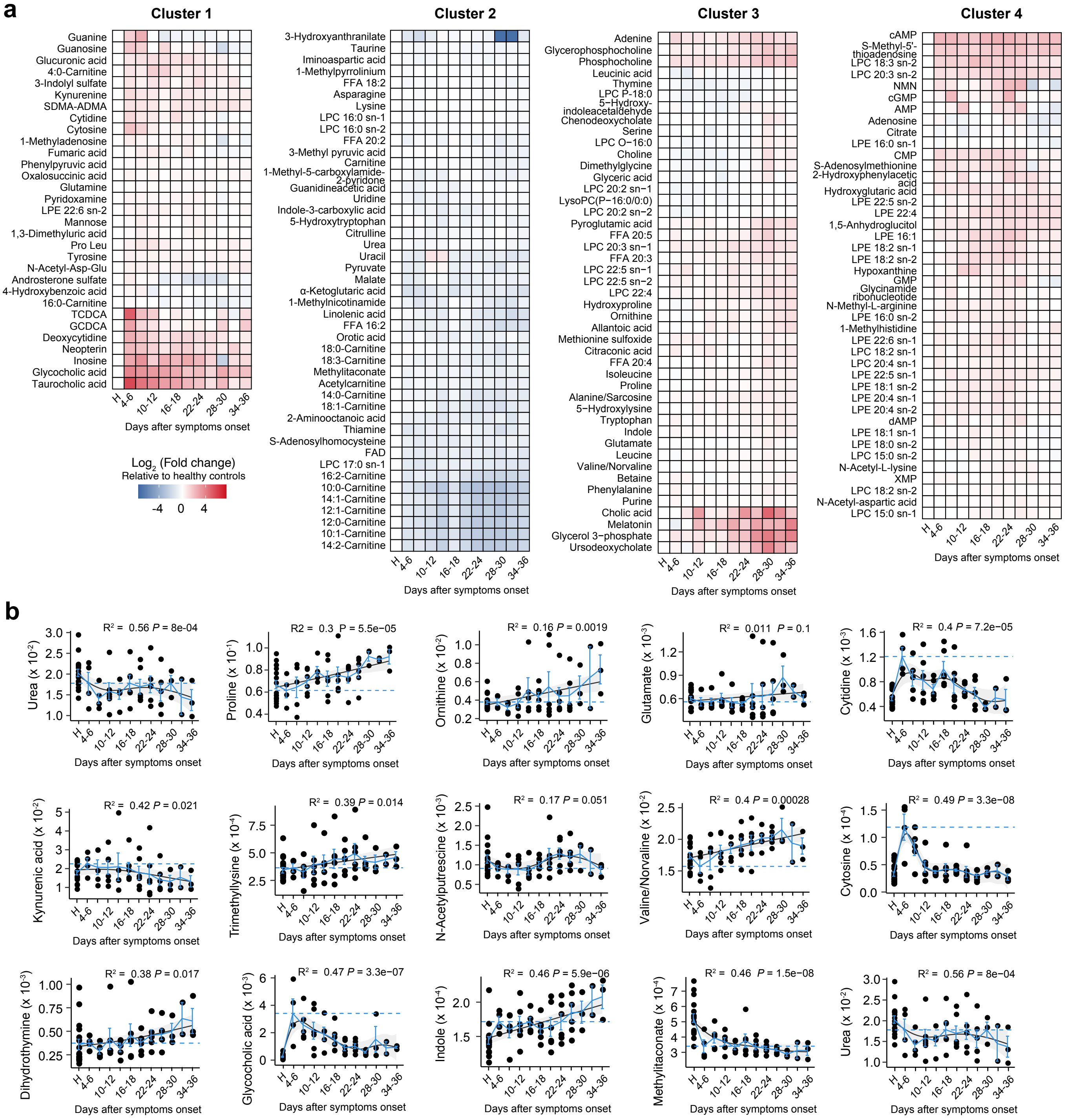


**Supplementary Fig. 6 Longitudinal metabolite trajectories in follow-up mild COVID-19 patients. a,** Heatmap comparison of metabolites at distinct time point in follow-up patients (n = 7) in each cluster. Log2 transformed fold change of metabolite abundance in each interval relative to healthy controls is represented by color intensity. **b,** Serum metabolite trajectories based on normalized data in follow-up patients (n = 7). Blue solid lines pass through the mean of each measurement at the specific time interval, and dotted lines represent the mean of measurements in healthy controls. GAM regression lines are represented by the black solid lines, with 95% confidence intervals for the regression donated by gray filled areas. *P* value was assessed by one-way ANOVA. Data are presented as mean ± SEM. with individual data points shown.

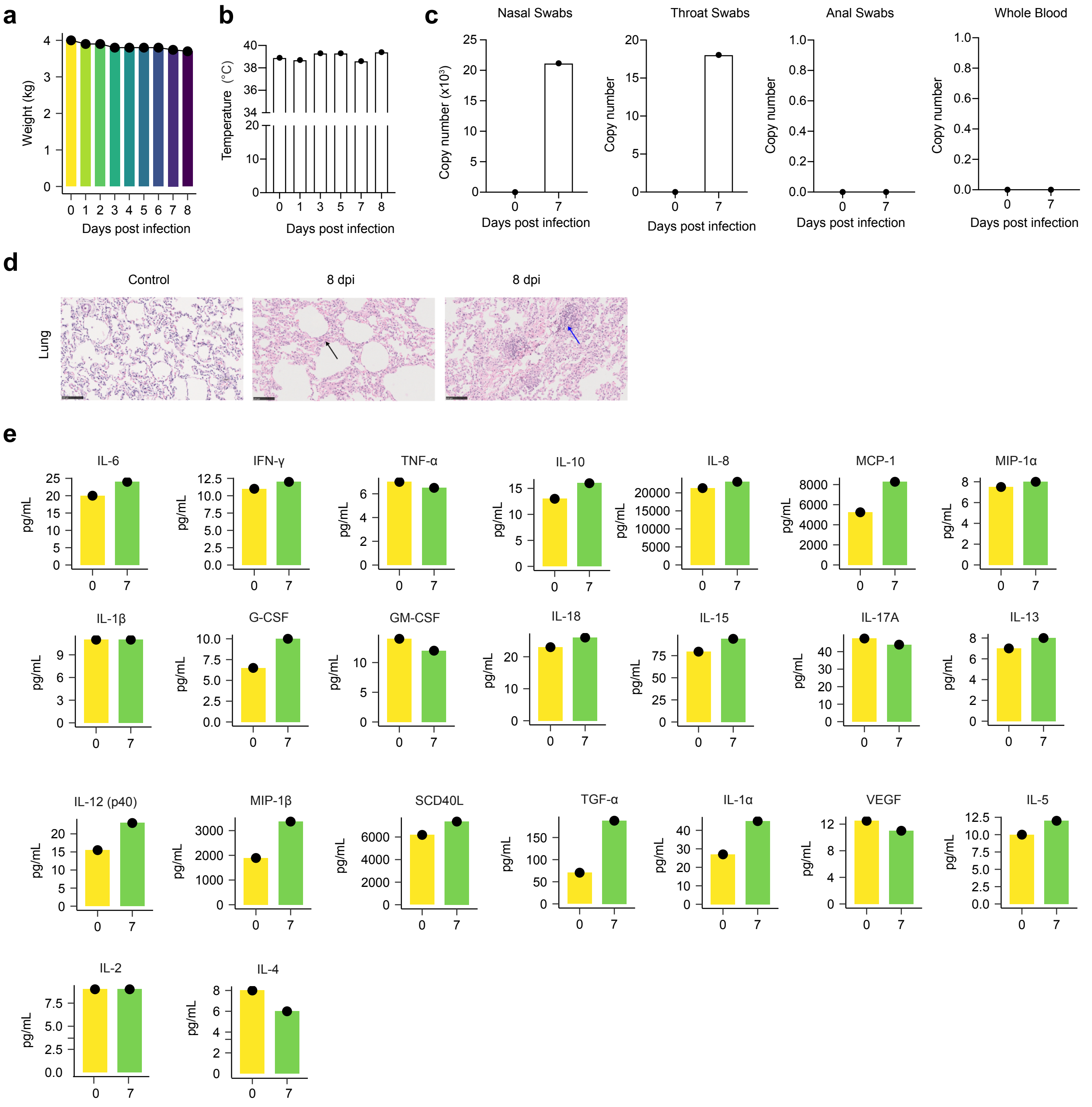


**Supplementary Fig 7. Experimental parameters of the SARS-CoV-2-infected rhesus macaque. a-c,** Body weight **(a)**, body temperature **(b)**, and virus copy number in swabs **(c)** of the rhesus macaque tested on day 0 and different days post SARS-CoV-2 infection. **d,** Hematoxylin and eosin (HE) staining of lung tissue in mock-infected and SARS-CoV-2-infected rhesus macaques. Alveolar thickening (black arrow), inflammatory cells infiltration (green arrow). Representative image of two or three independent experiments with similar results. **e,** Serum cytokine abundance on day 0 and day 7 post SARS-CoV-2 infection.

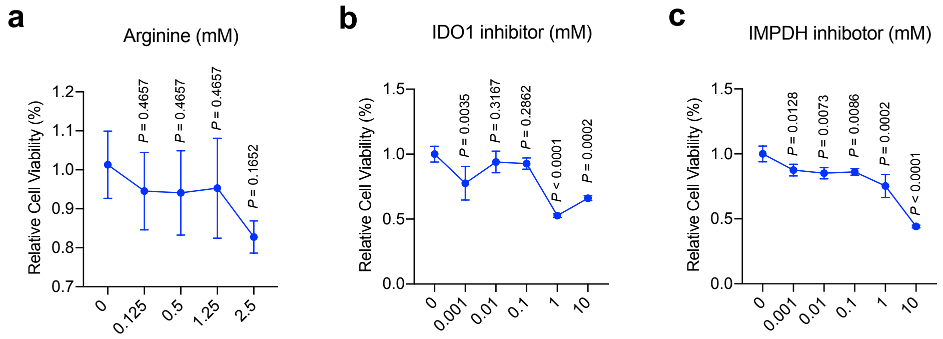


**Supplementary Fig. 8 Cell viability for compounds tested in Vero E6 cells. a-c,** Relative cell viability of Vero E6 cells (n = 3) with gradient concentration of arginine **(a)**, IDO1 inhibitor Epacadostat **(b)**, and IMPDH inhibitor MPA **(c)**. One-way ANOVA followed by BH multiple comparison test. Data are presented as mean ± SEM.

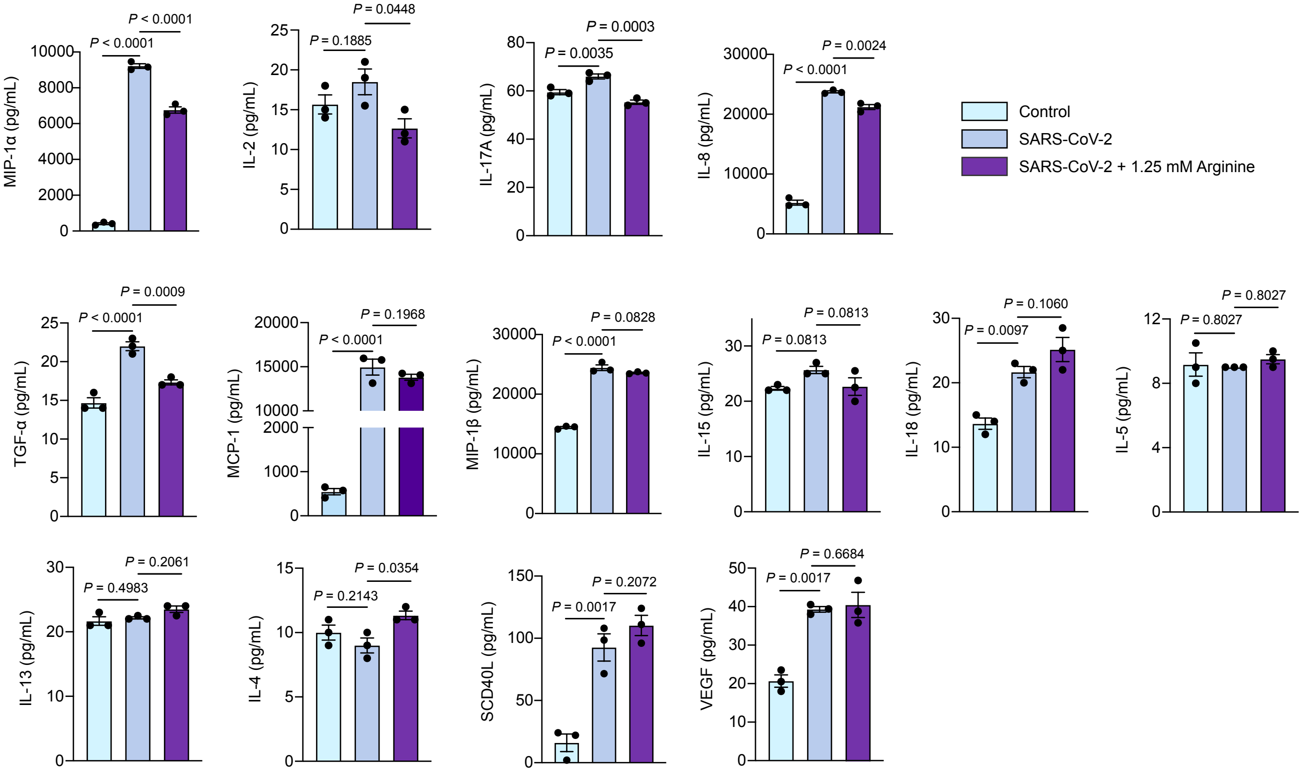


**Supplementary Fig. 9 Cytokine abundance after supplementation with arginine.** Levels of indicated cytokines and chemokines measured 24 h after supplementation of 1.25 mM arginine in PBMCs (n = 3) derived from mock-infected or SARS-CoV-2-infected rhesus macaques. One-way ANOVA followed by BH multiple comparison test. Data are presented as mean ± SEM. with individual data points shown.

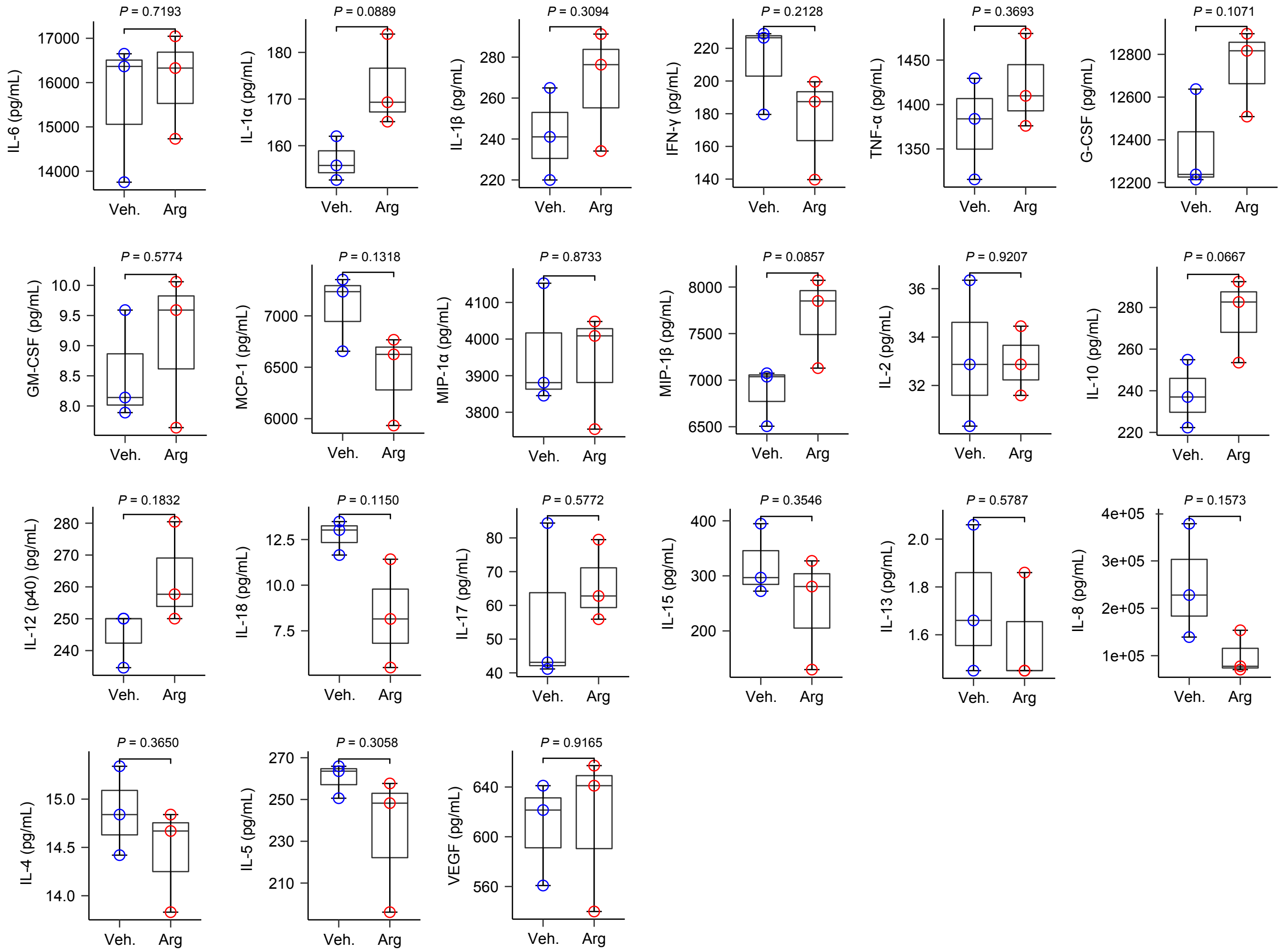


**Supplementary Fig. 10 Cytokine abundance after supplementation with arginine in normal PBMCs.** Levels of indicated cytokines and chemokines measured 24 h after supplementation with 1.25 mM arginine in PBMCs (n = 3) isolated from healthy controls. *P* value was assessed by unpaired, two-sided *t* test. For boxplots, centre is drawn through the median of the measurement, while the lower and upper bounds of the box correspond to the first and third percentile. Whiskers beyond these points denote minimum and maximum of measurement. Individual data points are shown. Veh., vehicle. Arg, arginine.

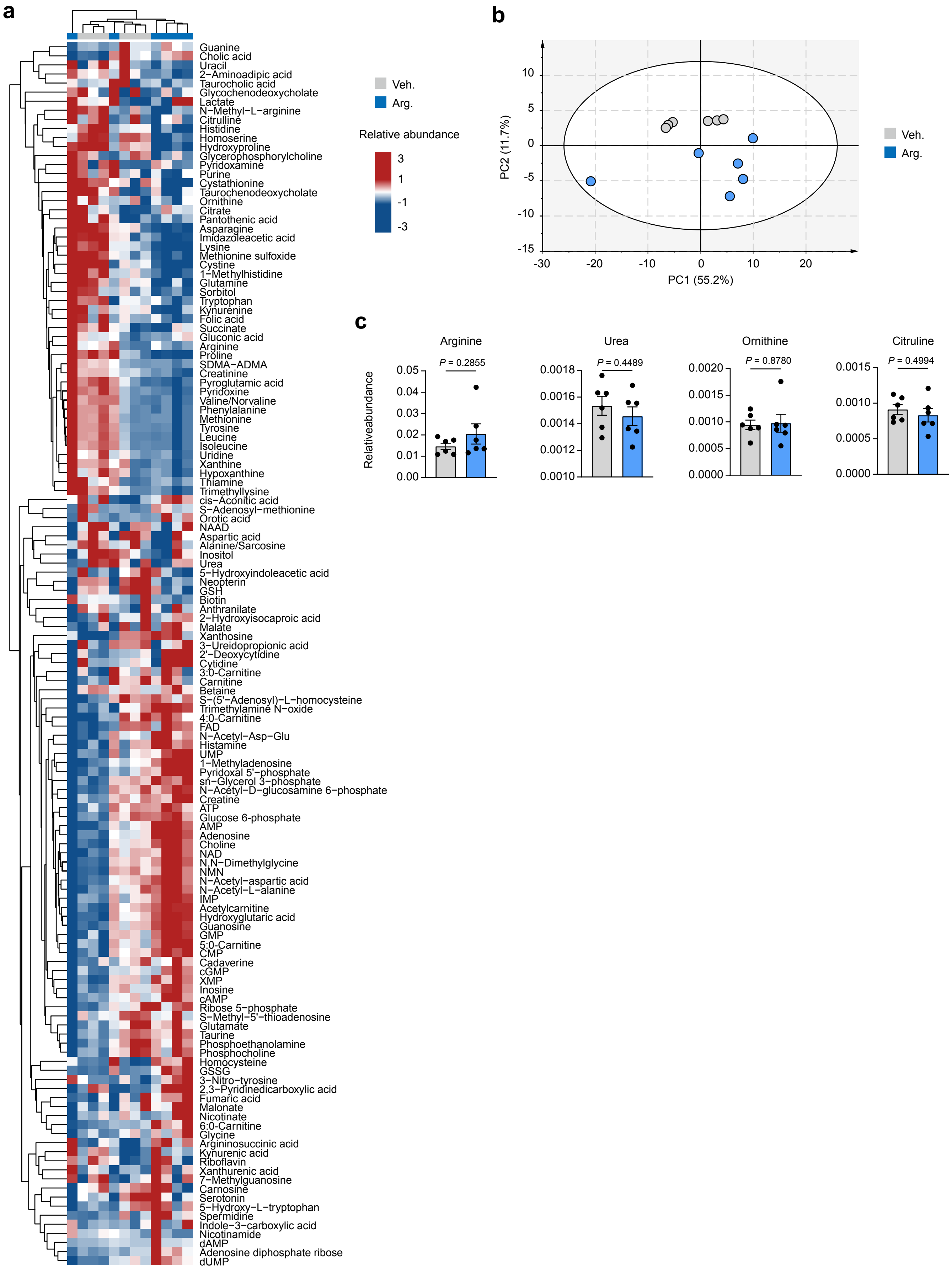
a
Veh.
Arg.
Relative abundance
3
1
-1
-3
Guanine
Cholic acid
Uracil
2-Aminoadipic acid
Taurocholic acid
Glycochenodeoxycholate
Lactate
N-Methyl-L-arginine
Citrulline
Histidine
Homoserine
Hydroxyproline
Glycerophosphorylcholine
Pyridoxamine
Purine
Cystathionine
Taurochenodeoxycholate
Ornithine
Citrate
Pantothenic acid
Asparagine
Imidazoleacetic acid
Lysine
Methionine sulfoxide
Cystine
1-Methylhistidine
Glutamine
Sorbitol
Tryptophan
Kynurenine
Folic acid
Succinate
Gluconic acid
Arginine
Proline
SDMA-ADMA
Creatinine
Pyroglutamic acid
Pyridoxine
Valine/Norvaline
Phenylalanine
Methionine
Tyrosine
Leucine
Isoleucine
Uridine
Xanthine
Hypoxanthine
Thiamine
Trimethyllysine
cis-Aconitic acid
S-Adenosyl-methionine
Orotic acid
NAAD
Aspartic acid
Alanine/Sarcosine
Inositol
Urea
5-Hydroxyindoleacetic acid
Neopterin
GSH
Biotin
Anthranilate
2-Hydroxyisocaproic acid
Malate
Xanthosine
3-Ureidopropionic acid
2'-Deoxycytidine
Cytidine
3:0-Carnitine
Carnitine
Betaine
S-(5'-Adenosyl)-L-homocysteine
Trimethylamine N-oxide
4:0-Carnitine
FAD
N-Acetyl-Asp-Glu
Histamine
UMP
1-Methyladenosine
Pyridoxal 5'-phosphate
sn-Glycerol 3-phosphate
N-Acetyl-D-glucosamine 6-phosphate
Creatine
ATP
Glucose 6-phosphate
AMP
Adenosine
Choline
NAD
N,N-Dimethylglycine
NMN
N-Acetyl-aspartic acid
N-Acetyl-L-alanine
IMP
Acetylcarnitine
Hydroxyglutaric acid
Guanosine
GMP
5:0-Carnitine
CMP
Cadaverine
cGMP
XMP
Inosine
cAMP
Ribose 5-phosphate
S-Methyl-5'-thioadenosine
Glutamate
Taurine
Phosphoethanolamine
Phosphocholine
Homocysteine
GSSG
3-Nitro-tyrosine
2,3-Pyridinedicarboxylic acid
Fumaric acid
Malonate
Nicotinate
6:0-Carnitine
Glycine
Argininosuccinic acid
Kynurenic acid
Riboflavin
Xanthurenic acid
7-Methylguanosine
Carnosine
Serotonin
5-Hydroxy-L-tryptophan
Spermidine
Indole-3-carboxylic acid
Nicotinamide
dAMP
Adenosine diphosphate ribose
dUMP
b
PC2 (11.7%)
PC1 (55.2%)
10
5
0
-5
-10
-15
-30
-20
-10
0
10
20
Veh.
Arg.
c
Relativeabundance
Arginine
P = 0.2855
0.05
0.04
0.03
0.02
0.01
0.00
Urea
P = 0.4489
0.0018
0.0016
0.0014
0.0012
0.0010
Ornithine
P = 0.8780
0.0020
0.0015
0.0010
0.0005
0.0000
Citruline
P = 0.4994
0.0015
0.0010
0.0005
0.0000

**Supplementary Fig. 11 Targeted metabolomics data of PBMC pellets after treatment with arginine.** **a,** Heatmap comparing the targeted metabolomics in PBMC pellets after treating PBMCs (n = 6) isolated from healthy controls with vehicle (veh.) or 1.25 mM arginine for 24 h. Relative abundance of metabolites is represented by color intensity. **b,** PCA of targeted metabolomics data in PBMC pellets after treating PBMCs isolated from healthy controls with vehicle (veh.) or 1.25 mM arginine for 24 h. **c,** Relative abundance of metabolites involved in arginine metabolism pathway. *P* value was assessed by unpaired, two-sided *t* test. Data are presented as mean ± SEM. with individual data points shown. Veh., vehicle. Arg., arginine.

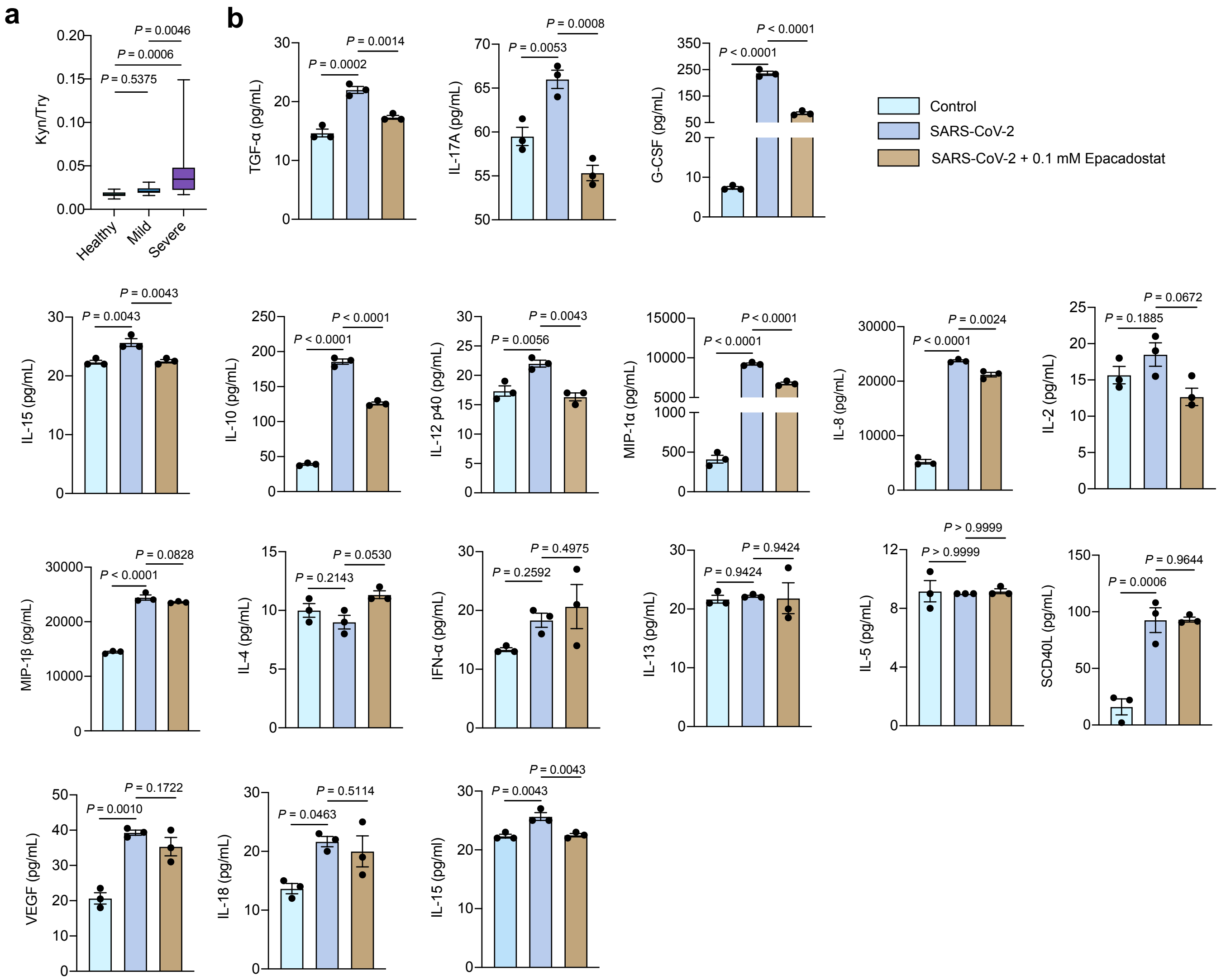

**Supplementary Fig. 12 Cytokine abundance after treatment with IDO1 inhibitor. a,** Ratio of kynurenine (Kyn) to tryptophan (Try) in healthy controls and COVID-19 patients. One-way ANOVA followed by BH multiple comparison test. For boxplots, centre is drawn through the median of the measurement, while the lower and upper bounds of the box correspond to the first and third percentile. Whiskers beyond these points denote minimum and maximum of measurement. **b,** Levels of indicated cytokines and chemokines measured 24 h after 0.1 mM IDO1 inhibitor Epacadostat in PBMCs (n = 3) derived from mock-infected or SARS-CoV-2-infected rhesus macaques. One-way ANOVA followed by BH multiple comparison test. Data are presented as mean ± SEM with individual data points shown.

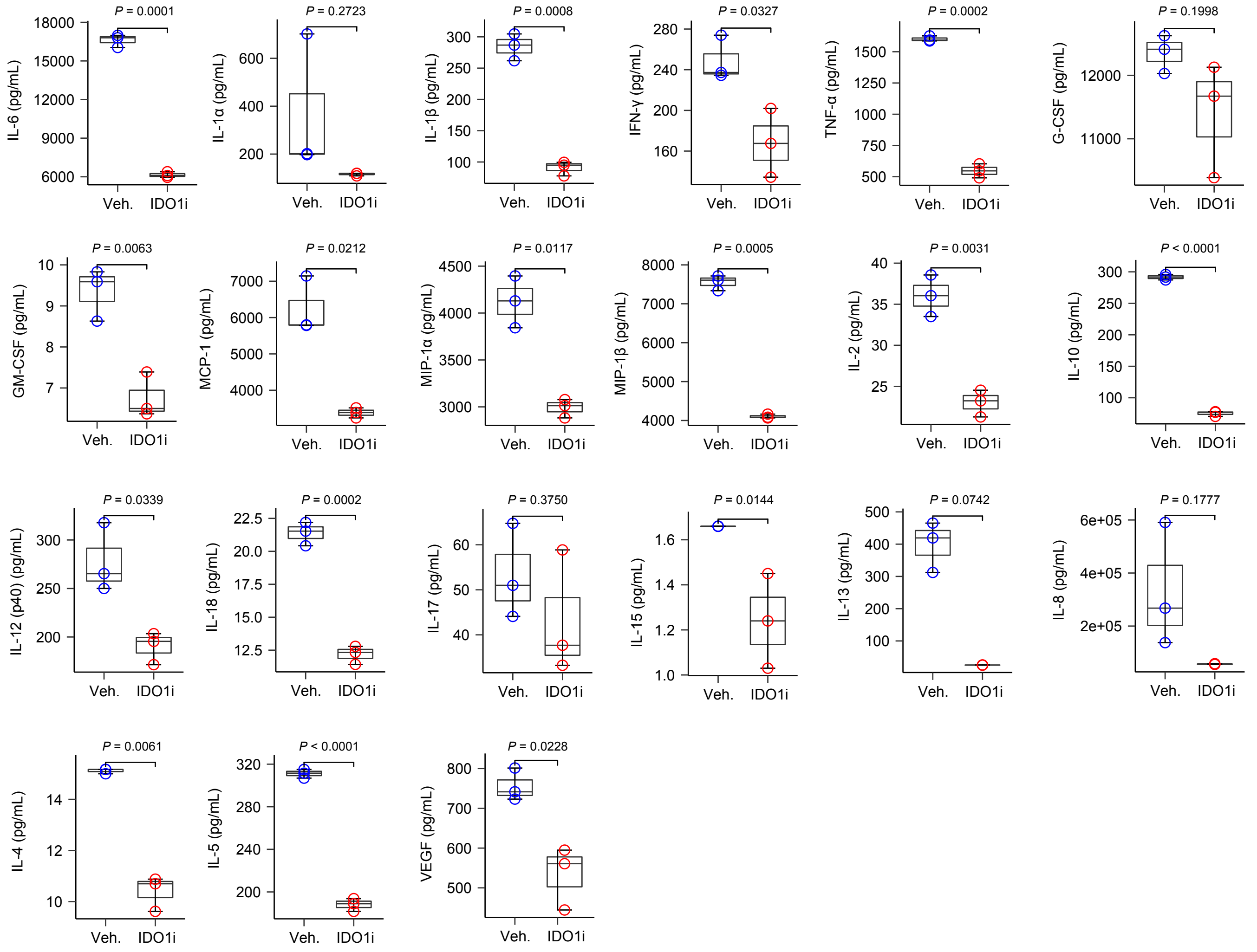


**Supplementary Fig. 13 Cytokine abundance after treatment with IDO1 inhibitor in normal PBMCs.** Levels of indicated cytokines and chemokines measured 24 h after treatment with 0.1 mM IDO1 inhibitor Epacadostat in PBMCs (n = 3) isolated from healthy controls. *P* value was assessed by unpaired, two-sided *t* test. For boxplots, centre is drawn through the median of the measurement, while the lower and upper bounds of the box correspond to the first and third percentile. Whiskers beyond these points denote minimum and maximum of measurement. Individual data points are shown. Veh., vehicle. IDO1i, IDO1 inhibitor Epacadostat.

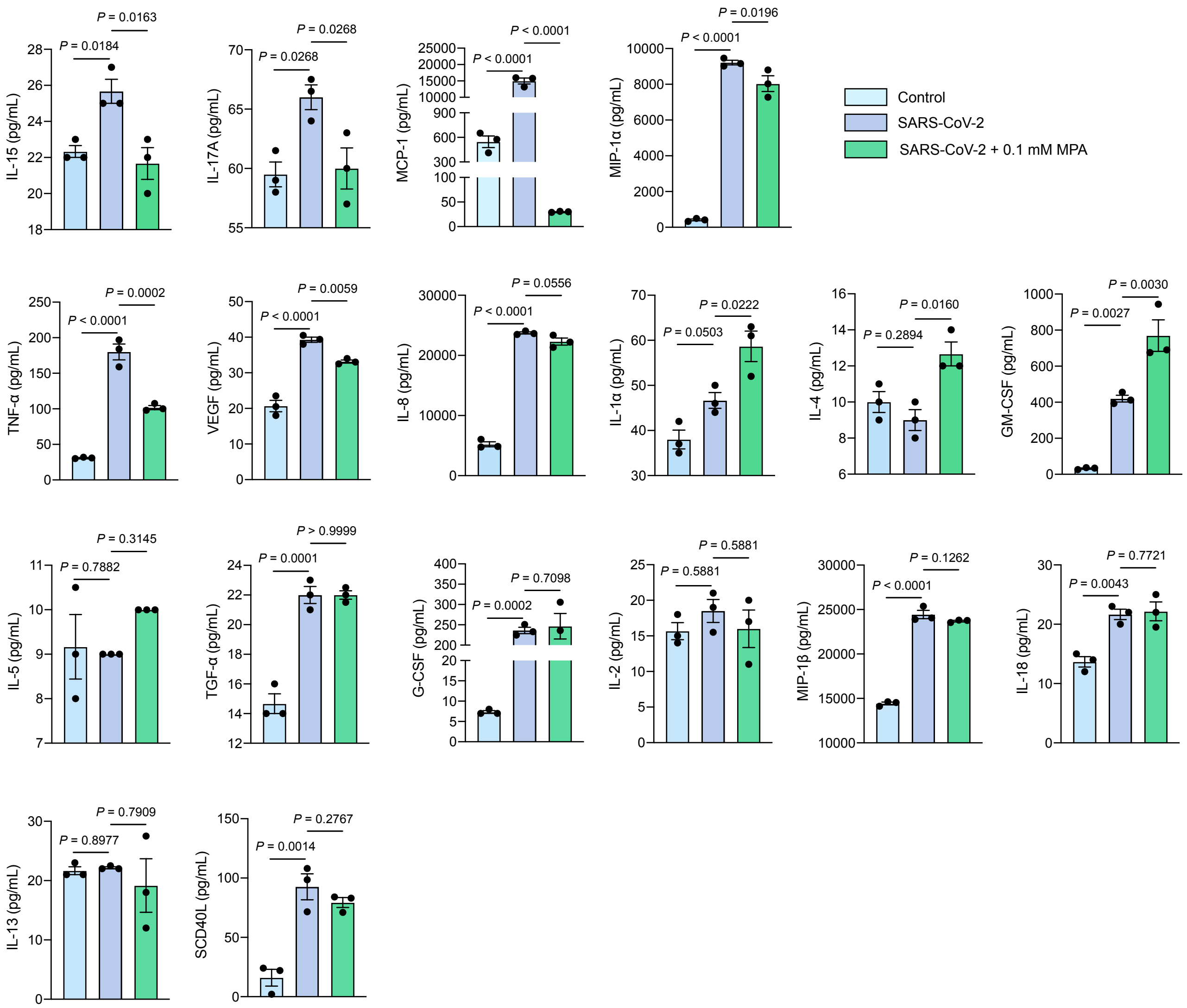


**Supplementary Fig. 14 Cytokine abundance after treatment with IMPDH inhibitor.** Levels of indicated cytokines and chemokines measured 24 h after treatment of 0.1 mM IMPDH inhibitor MPA in PBMCs (n = 3) derived from mock-infected or SARS-CoV-2-infected rhesus macaques. One-way ANOVA followed by BH multiple comparison test. Data are presented as mean ± SEM. with individual data points shown.

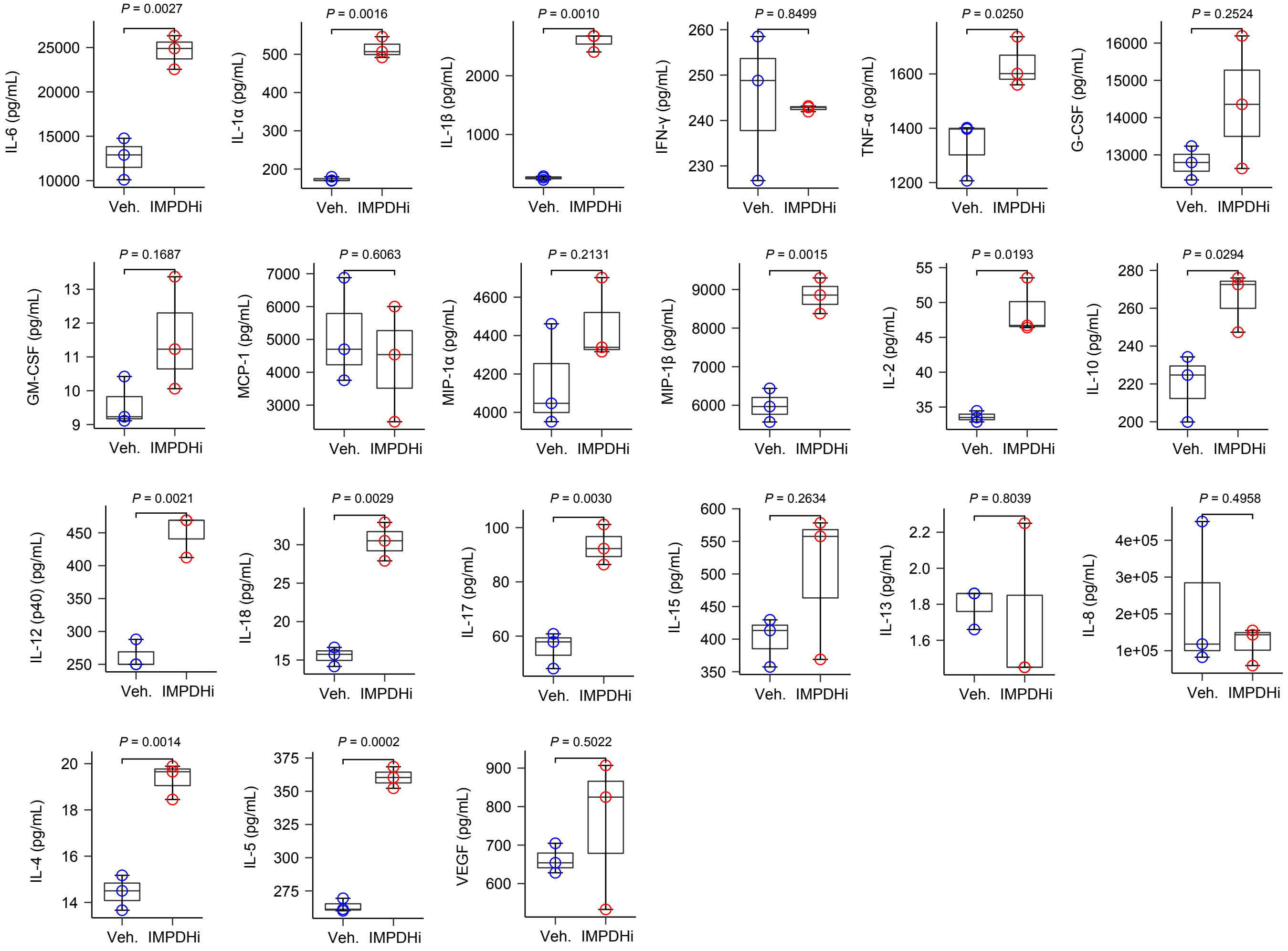


**Supplementary Fig. 15 Cytokine abundance after treatment with IMPDH inhibitor in normal PBMCs.** Levels of indicated cytokines and chemokines measured 24 h after treatment with 0.1 mM IMPDH inhibitor mycophenolic acid (MPA) in PBMCs (n = 3) isolated from healthy controls. *P* value was assessed by unpaired, two-sided *t* test. For boxplots, centre is drawn through the median of the measurement, while the lower and upper bounds of the box correspond to the first and third percentile. Whiskers beyond these points denote minimum and maximum of measurement. Individual data points are shown. Veh., vehicle. IMPDHi, IMPDH inhibitor MPA.

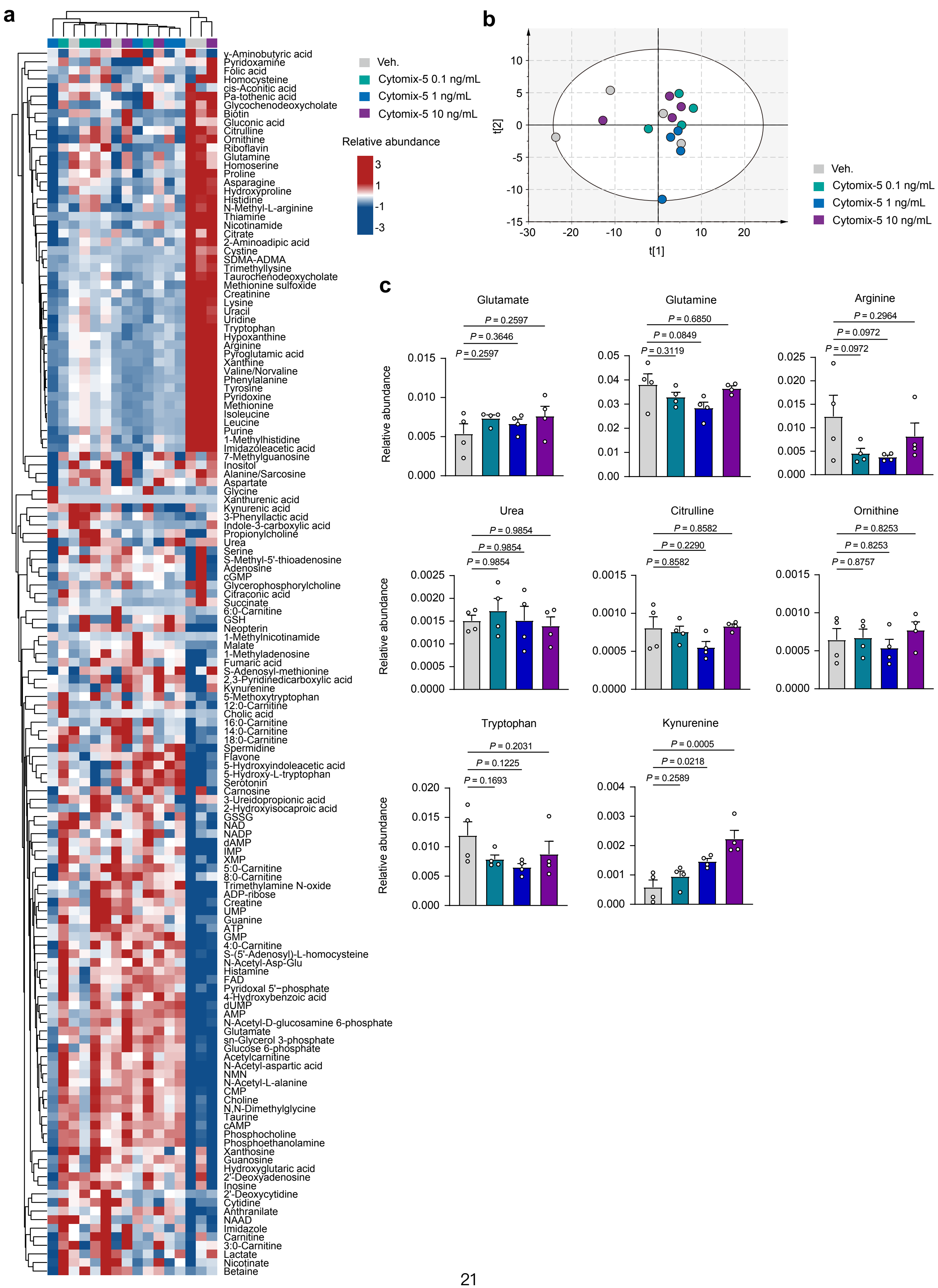
a
γ-Aminobutyric acid
Pyridoxamine
Folic acid
Homocysteine
cis-Aconitic acid
Pa-tothenic acid
Glycochenodeoxycholate
Biotin
Gluconic acid
Citrulline
Ornithine
Riboflavin
Glutamine
Homoserine
Proline
Asparagine
Hydroxyproline
Histidine
N-Methyl-L-arginine
Thiamine
Nicotinamide
Citrate
2-Aminoadipic acid
Cystine
SDMA-ADMA
Trimethyllysine
Taurochenodeoxycholate
Methionine sulfoxide
Creatinine
Lysine
Uracil
Uridine
Tryptophan
Hypoxanthine
Arginine
Pyroglutamic acid
Xanthine
Valine/Norvaline
Phenylalanine
Tyrosine
Pyridoxine
Methionine
Isoleucine
Leucine
Purine
1-Methylhistidine
Imidazoleacetic acid
7-Methylguanosine
Inositol
Alanine/Sarcosine
Aspartate
Glycine
Xanthurenic acid
Kynurenic acid
3-Phenyllactic acid
Indole-3-carboxylic acid
Propionylcholine
Urea
Serine
S-Methyl-5'-thioadenosine
Adenosine
cGMP
Glycerophosphorylcholine
Citraconic acid
Succinate
6:0-Carnitine
GSH
Neopterin
1-Methylnicotinamide
Malate
1-Methyladenosine
Fumaric acid
S-Adenosyl-methionine
2,3-Pyridinedicarboxylic acid
Kynurenine
5-Methoxytryptophan
12:0-Carnitine
Cholic acid
16:0-Carnitine
14:0-Carnitine
18:0-Carnitine
Spermidine
Flavone
5-Hydroxyindoleacetic acid
5-Hydroxy-L-tryptophan
Serotonin
Carnosine
3-Ureidopropionic acid
2-Hydroxyisocaproic acid
GSSG
NAD
NADP
dAMP
IMP
XMP
5:0-Carnitine
8:0-Carnitine
Trimethylamine N-oxide
ADP-ribose
Creatine
UMP
Guanine
ATP
GMP
4:0-Carnitine
S-(5'-Adenosyl)-L-homocysteine
N-Acetyl-Asp-Glu
Histamine
FAD
Pyridoxal 5'−phosphate
4-Hydroxybenzoic acid
dUMP
AMP
N-Acetyl-D-glucosamine 6-phosphate
Glutamate
sn-Glycerol 3-phosphate
Glucose 6-phosphate
Acetylcarnitine
N-Acetyl-aspartic acid
NMN
N-Acetyl-L-alanine
CMP
Choline
N,N-Dimethylglycine
Taurine
cAMP
Phosphocholine
Phosphoethanolamine
Xanthosine
Guanosine
Hydroxyglutaric acid
2'-Deoxyadenosine
Inosine
2'-Deoxycytidine
Cytidine
Anthranilate
NAAD
Imidazole
Carnitine
3:0-Carnitine
Lactate
Nicotinate
Betaine
Veh.
Cytomix-5 0.1 ng/mL
Cytomix-5 1 ng/mL
Cytomix-5 10 ng/mL
Relative abundance
3
1
-1
-3
b
t[2]
t[1]
10
5
0
-5
-10
-15
-30
-20
-10
0
10
20
Veh.
Cytomix-5 0.1 ng/mL
Cytomix-5 1 ng/mL
Cytomix-5 10 ng/mL
c
Glutamate
P = 0.2597
P = 0.3646
P = 0.2597
Relative abundance
0.015
0.010
0.005
0.000
Glutamine
P = 0.6850
P = 0.0849
P = 0.3119
0.05
0.04
0.03
0.02
0.01
0.00
Arginine
P = 0.2964
P = 0.0972
P = 0.0972
0.025
0.020
0.015
0.010
0.005
0.000
Urea
P = 0.9854
P = 0.9854
P = 0.9854
Relative abundance
0.0025
0.0020
0.0015
0.0010
0.0005
0.0000
Citrulline
P = 0.8582
P = 0.2290
P = 0.8582
0.0015
0.0010
0.0005
0.0000
Ornithine
P = 0.8253
P = 0.8253
P = 0.8757
0.0015
0.0010
0.0005
0.0000
Tryptophan
P = 0.2031
P = 0.1225
P = 0.1693
Relative abundance
0.020
0.015
0.010
0.005
0.000
Kynurenine
P = 0.0005
P = 0.0218
P = 0.2589
0.004
0.003
0.002
0.001
0.000

**Supplementary Fig. 16 Targeted metabolomics of PBMC pellets after treatment with the cytokine cocktail. a,** Heatmap comparing the targeted metabolomics in PBMC pellets (n = 4) after treating PBMCs isolated from healthy controls with vehicle (veh.) or cytokine mixtures of IL-6, IL-1α, IL-1β, IFN-γ, and TNF-α (cytomix-5) with indicated concentrations for 24 h. Relative abundance of metabolites is represented by color intensity. **b,** PCA of targeted metabolomics data in PBMC pellets after treating PBMCs isolated from healthy controls with vehicle (veh.) or cytomix-5 with indicated concentrations for 24 h. **c,** Relative abundance of metabolite involved in arginine and tryptophan metabolism pathways in PBMC pellets (n = 4) after treating PBMCs isolated from healthy controls with vehicle (veh.) or cytomix-5 with indicated concentrations for 24 h. One-way ANOVA followed by BH multiple comparison test. Data are presented as mean ± SEM. with individual data points shown.

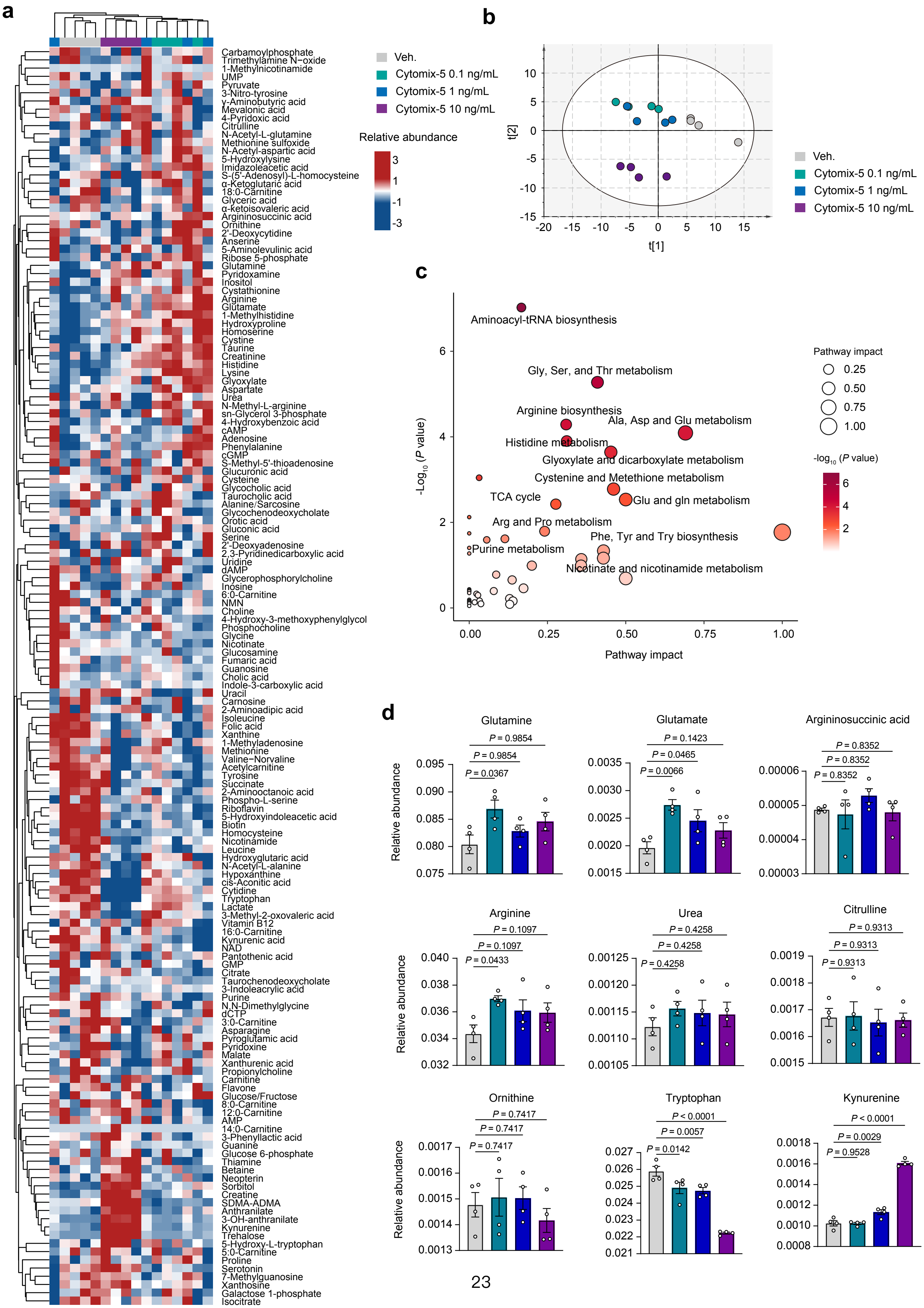
a
Relative abundance
3
1
-1
-3
Veh.
Cytomix-5 0.1 ng/mL
Cytomix-5 1 ng/mL
Cytomix-5 10 ng/mL
Carbamoylphosphate
Trimethylamine N−oxide
1-Methylnicotinamide
UMP
Pyruvate
3-Nitro-tyrosine
γ-Aminobutyric acid
Mevalonic acid
4-Pyridoxic acid
Citrulline
N-Acetyl-L-glutamine
Methionine sulfoxide
N-Acetyl-aspartic acid
5-Hydroxylysine
Imidazoleacetic acid
S-(5'-Adenosyl)-L-homocysteine
α-Ketoglutaric acid
18:0-Carnitine
Glyceric acid
α-ketoisovaleric acid
Argininosuccinic acid
Ornithine
2'-Deoxycytidine
Anserine
5-Aminolevulinic acid
Ribose 5-phosphate
Glutamine
Pyridoxamine
Inositol
Cystathionine
Arginine
Glutamate
1-Methylhistidine
Hydroxyproline
Homoserine
Cystine
Taurine
Creatinine
Histidine
Lysine
Glyoxylate
Aspartate
Urea
N-Methyl-L-arginine
sn-Glycerol 3-phosphate
4-Hydroxybenzoic acid
cAMP
Adenosine
Phenylalanine
cGMP
S-Methyl-5'-thioadenosine
Glucuronic acid
Cysteine
Glycocholic acid
Taurocholic acid
Alanine/Sarcosine
Glycochenodeoxycholate
Orotic acid
Gluconic acid
Serine
2'-Deoxyadenosine
2,3-Pyridinedicarboxylic acid
Uridine
dAMP
Glycerophosphorylcholine
Inosine
6:0-Carnitine
NMN
Choline
4-Hydroxy-3-methoxyphenylglycol
Phosphocholine
Glycine
Nicotinate
Glucosamine
Fumaric acid
Guanosine
Cholic acid
Indole-3-carboxylic acid
Uracil
Carnosine
2-Aminoadipic acid
Isoleucine
Folic acid
Xanthine
1-Methyladenosine
Methionine
Valine−Norvaline
Acetylcarnitine
Tyrosine
Succinate
2-Aminooctanoic acid
Phospho-L-serine
Riboflavin
5-Hydroxyindoleacetic acid
Biotin
Homocysteine
Nicotinamide
Leucine
Hydroxyglutaric acid
N-Acetyl-L-alanine
Hypoxanthine
cis-Aconitic acid
Cytidine
Tryptophan
Lactate
3-Methyl-2-oxovaleric acid
Vitamin B12
16:0-Carnitine
Kynurenic acid
NAD
Pantothenic acid
GMP
Citrate
Taurochenodeoxycholate
3-Indoleacrylic acid
Purine
N,N-Dimethylglycine
dCTP
3:0-Carnitine
Asparagine
Pyroglutamic acid
Pyridoxine
Malate
Xanthurenic acid
Propionylcholine
Carnitine
Flavone
Glucose/Fructose
8:0-Carnitine
12:0-Carnitine
AMP
14:0-Carnitine
3-Phenyllactic acid
Guanine
Glucose 6-phosphate
Thiamine
Betaine
Neopterin
Sorbitol
Creatine
SDMA-ADMA
Anthranilate
3-OH-anthranilate
Kynurenine
Trehalose
5-Hydroxy-L-tryptophan
5:0-Carnitine
Proline
Serotonin
7-Methylguanosine
Xanthosine
Galactose 1-phosphate
Isocitrate
b
t[2]
t[1]
Veh.
Cytomix-5 0.1 ng/mL
Cytomix-5 1 ng/mL
Cytomix-5 10 ng/mL
c
-Log10 (P value)
Pathway impact
Aminoacyl-tRNA biosynthesis
Gly, Ser, and Thr metabolism
Arginine biosynthesis
Ala, Asp and Glu metabolism
Histidine metabolism
Glyoxylate and dicarboxylate metabolism
Cystenine and Metethione metabolism
TCA cycle
Glu and gln metabolism
Arg and Pro metabolism
Phe, Tyr and Try biosynthesis
Purine metabolism
Nicotinate and nicotinamide metabolism
Pathway impact
0.25
0.50
0.75
1.00
-log10 (P value)
d
Relative abundance
Glutamine
P = 0.9854
P = 0.9854
P = 0.0367
Glutamate
P = 0.1423
P = 0.0465
P = 0.0066
Argininosuccinic acid
P = 0.8352
P = 0.8352
P = 0.8352
Arginine
P = 0.1097
P = 0.1097
P = 0.0433
Urea
P = 0.4258
P = 0.4258
P = 0.4258
Citrulline
P = 0.9313
P = 0.9313
P = 0.9313
Ornithine
P = 0.7417
P = 0.7417
P = 0.7417
Tryptophan
P < 0.0001
P = 0.0057
P = 0.0142
Kynurenine
P < 0.0001
P = 0.0029
P = 0.9528

**Supplementary Fig. 17 Targeted metabolomics of PBMC culture media after treatment with the cytokine cocktail. a,** Heatmap comparing the targeted metabolomics in PBMC culture media (n = 4) after treating PBMCs isolated from healthy controls with vehicle (veh.) or cytomix-5 with indicated concentrations for 24 h. Relative abundance of metabolites is represented by color intensity. **b,** PCA of targeted metabolomics data in PBMC culture media after treating PBMCs isolated from healthy controls with vehicle (veh.) or cytomix-5 with indicated concentrations for 24 h. **c,** Pathway impact analysis of metabolites with Variable Importance for the Projection (VIP) score > 1 in PBMC culture media after treating PBMCs with cytomix-5 with indicated concentrations for 24 h. Significance is represented by color intensity, and size represents pathway impact. **d,** Relative abundance of metabolite involved in arginine, tryptophan metabolism pathways in PBMC pellets (n = 4) after treating PBMCs isolated from healthy controls with vehicle (veh.) or cytomix-5 with indicated concentrations for 24 h. One-way ANOVA followed by BH multiple comparison test. Data are presented as mean ± SEM. with individual data points shown.

## Supplementary Tables

### Supplementary Table 1. Summarized clinical characteristics of healthy controls, non-COVID-19 acute upper respiratory tract infection patients and COVID-19 patients

| | **Healthy Control** | **Non-COVID-19** | **COVID-19** | | |
|---|---|---|---|---|---|
| Variables | Healthy Control (n = 17) | Non-COVID-19 (n = 20) | Mild (n = 14) | Severe (n = 23) | Follow-up (n = 7) |
| Sex - no. (%) [a] | | | | | |
| Male | 8 (47.1%) | 10 (50%) | 8 (57.1%) | 9 (39.1%) | 4 (57.1%) |
| Female | 9 (52.9%) | 10 (50%) | 6 (42.9%) | 14 (60.9%) | 3 (42.9%) |
| Age - year | | | | | |
| Mean ± SD. [b] | 47.5 ± 13.8 | 43.5 ± 17.6 | 34.2 ± 19.0 | 60.8 ± 16.7 | 41.9 ± 19.3 |
| Median (IQR) [c] | 50 (40-54) | 42 (30-60) | 38 (20-48) | 65 (52-78) | 46 (28-56) |
| Range | 20-72 | 15-68 | 0.66-67 | 31-80 | 16-63 |
| BMI, kg/m2 | | | | | |
| Median (IQR) | 25.7 (24.7-27.4) | - | 23.4 (20.7-26.1) | 24.5 (23.0-27.2) | - |
| Time from Onset to Admission, Days | | | | | |
| Median (IQR) | - | - | 6 (4-8) | 7 (5-8) | 2 (1-3) |
| Symptoms - no. (%) | | | | | |
| Fever | - | 11 (55%) | 11 (78.6%) | 13 (56.5%) | 5 (71.4%) |
| Fatigue | - | 1 (5%) | 1 (7.1%) | 12 (52.2%) | 2 (28.6%) |
| Dry cough | - | 2 (10%) | 8 (57.1%) | 12 (52.2%) | 3 (42.9%) |
| Inappetence | - | - | 3 (21.4%) | 13 (56.5%) | - |
| Myalgia | - | - | 1 (7.1%) | 3 (13.0%) | 2 (28.6%) |
| Dyspnea | - | - | - | 9 (39.1%) | - |
| Expectoration | - | - | 2 (14.3%) | 9 (39.1%) | - |
| Pharyngalgia | - | 4 (20%) | 2 (14.3%) | 1 (4.3%) | 1 (14.3%) |
| Diarrhea | - | - | 3 (21.4%) | 2 (8.7%) | - |
| Nausea | - | - | - | 3 (13.0%) | - |
| Dizziness | - | - | - | 3 (13.0%) | - |
| Headache | - | 1 (5%) | 1 (7.1%) | 2 (8.7%) | - |
| Abdominal pain | - | - | - | 2 (8.7%) | - |
| Chill | - | 2 (10%) | 1 (7.1%) | 4 (17.4%) | - |
| Rhinorrhea | - | - | 3 (21.4%) | 3 (13.0%) | - |
| Chest stuffiness | - | - | 3 (21.4%) | 9 (39.1%) | - |
| Nasal congestion | - | 4 (20%) | 1 (7.1%) | 2 (8.7%) | - |

| | | | | | |
|---|---|---|---|---|---|
| Comorbidity- no. (%) | | | | | |
| Hypertension | - | - | - | 3 (13.0%) | 1 (14.3%) |
| Cardiovascular disease | - | - | - | 1 (4.3%) | - |
| Diabetes | - | - | - | 2 (8.7%) | - |
| COPD | - | - | - | 1 (4.3%) | - |
| Chronic liver disease | - | - | 1 (7.1%) | 1 (4.3%) | - |
| Oxygenation Index - mmHg | | | | | |
| Median (IQR) | - | - | 390 (362-395) | 276 (160-340) | 429 (384-450) |
| Treatment - no. (%) | | | | | |
| Kaletra | - | - | 13 (92.9%) | 23 (100.0%) | 7 (100.0%) |
| Oseltamivir | - | - | 1 (7.1%) | 3 (13.0%) | - |
| Interferon therapy | - | - | 14 (100.0%) | 23 (100.0%) | 7 (100.0%) |
| Antibiotic therapy | - | - | 1 (7.1%) | 18 (78.3%) | 2 (28.6%) |
| Glucocorticoid | - | - | - | 10 (43.5%) | 2 (28.6%) |
| Chinese medicine therapy | - | - | 3 (21.4%) | 17 (73.9%) | 7 (100.0%) |
| Oxygen therapy | - | - | - | 9 (39.1%) | 7 (100.0%) |
| Mechanical ventilation | - | - | 13 (92.9%) | 23 (100.0%) | - |
| Blood routine | | | | | |
| White blood cell count, × $10^9$/L | - | - | - | 9.6 (9.3-13.8) | 4.7 (4.3-6.1) |
| Red blood cell count, × $10^{12}$/L | - | - | - | 4.1 (3.8-4.1) | 4.9 (4.4-5.3) |
| Neutrophils count, × $10^9$/L | - | - | 2.7 (2.1-3.2) | 3.4 (3.0-4.9) | 2.7 (2.4-3.7) |
| Lymphocytes count, × $10^9$/L | - | - | 1.5 (1.2-2.1) | 0.8 (0.6-1.1) | 1.2 (1.1-1.4) |
| Platelets count, × $10^9$/L | - | - | - | 259.0 (163.0-356.0) | 179 (158-185.5) |
| Haemoglobin, g/L | - | - | 30.8 (30.3-31.5) | 29.6 (28.8-30.7) | - |
| Blood biochemistry | | | | | |
| Alanine aminotransferase, U/L | 17.0 (15.0-22.0) | 104 (7-1591) | 16.6 (13.8-33.7) | 37.3 (18.2-45.0) | 28.0 (21.0-29.5) |
| Aspartate aminotransferase, U/L | 19.0 (17.0-24.0) | 50.0 (13-549) | 20.9 (17.4-26.3) | 30.0 (20.4-39.1) | 26.0 (22.5-27.0) |
| γ-glutamyl transferase, U/L | 18.5 (17.8-20.5) | 42.3 (14-116) | 24.0 (14.0-44.0) | 49.0 (24.0-62.0) | 23.0 (19.0-38.5) |
| Total bilirubin, μmol/L | 11.8 (9.4-14.6) | 13.2 (4-54.7) | 8.9 (5.2-11.3) | 14.8 (8.4-19.8) | 7.7 (5.2-11.4) |
| Direct bilirubin, μmol/L | 1.4 (1.0-2.2) | 4.9 (1.1-32.5) | 4.5 (3.0-5.0) | 5.4 (4.1-12.9) | 2.1 (1.3-2.7) |
| Indirect bilirubin, μmol/L | 7.2 (5.8-8.5) | 8.3 (3.1-22.6) | 4.3 (2.5-6.4) | 6.3 (5.1-7.6) | - |
| Lactate dehydrogenase, U/L | - | - | 208.0 (157.0-226.0) | 340.0 (242.0-381.0) | 139.0 (123.2-151.0) |
| C-reactive protein, mg/L | - | - | 2.5 (1.2-7.6) | 67.3 (14.2-121.6) | 1.2 (0.8-8.2) |
| Procalcitonin, ng/ml | - | - | 0.032 (0.031-0.043) | 0.078 (0.051-0.154) | 0.050 (0.050-0.070) |

| | | | | | |
|---|---|---|---|---|---|
| Creatinine, μmol/L | 54.0 (43.0-59.0) | 56.3 (25-87.6) | 70.0 (63.0-74.0) | 61.0 (50.0-64.0) | 63.0 (53.0-82.0) |
| Urea, mmol/L | 5.3 (4.4-6.1) | 5.1 (3.54-6.85) | 3.5 (3.2-4.4) | 3.8 (2.8-5.2) | 5.0 (3.6-5.4) |
| Uric acid, μmol/L | 326.0 (264.0-373.0) | 364.6 (211.3-531.9) | - | 151.5 (106.0-232.5) | 269.0 (225.5-389.5) |
| Glucose, mol/L | 5.0 (4.9-5.5) | 5.1 (4.2-7.9) | 5.5 (4.9-5.8) | 6.9 (5.4-11.8) | 7.3 (5.5-8.3) |
| Total protein, g/L | 72.0 (70.2-73.8) | 75.8 (64.6-83.6) | 67.1 (64.5-68.5) | 62.6 (59.2-66.7) | 70.3 (64.3-73.5) |
| Albumin, g/L | 45.1 (43.3-46.5) | 44.1 (32.4-51.5) | 40.8 (38.8-41.6) | 32.9 (31.4-36.7) | - |
| Globulin, g/L | 27.0 (24.1-28.4) | 31.7 (25.5-41.3) | 26.0 (24.0-29.0) | 30.0 (26.0-32.0) | 26.3 (25.6-27.8) |
| Triglyceride, mmol/L | 1.4 (1.1-2.0) | 1.3 (0.6-2.6) | - | 1.3 (0.9-2.0) | 1.1 (1.0-1.4) |
| Total cholesterol, mmol/L | 4.9 (4.5-5.9) | 4.5 (1.8-6.8) | - | 3.5 (3.0-3.8) | 4.2 (3.8-4.4) |
| High density lipoprotein, mmol/L | 1.3 (1.1-1.5) | 1.2 (0.5-1.5) | - | 0.9 (0.8-1.1) | 1.2 (1.2-1.4) |
| Low density lipoprotein, mmol/L | 3.0 (2.4-3.5) | 2.8 (0.9-4.5) | - | 2.2 (1.8-2.3) | 2.2 (1.8-2.2) |

[a]no. (%): number.

[b]SD: Standard Deviation.

[c]IQR: Interquartile range.

## Supplementary Table 2. Primers of RT-PCR testing for SARS-CoV-2

| Gene | Strand | Primer sequence (5'-3') |
|---|---|---|
| ORF1ab | Sense | CCCTGTGGGTTTTACACTTA |
| | Antisense | ACGATTGTGCATCAGCTGA |
| | Probe | CCGTCTGCGGTATGTGGAAAGGTTATGG |
| N | Sense | GGGGAACTTCTCCTGCTAGAAT |
| | Antisense | CAGACATTTTGCTCTCAAGCTG |
| | Probe | TTGCTGCTGCTTGACAGATT |
