# Supplementary material for: Integrated cytokine and metabolite analysis reveals immunometabolic reprogramming in COVID-19 patients with therapeutic implications
Source: Nat Commun. 2021 Mar 12;12:1618. doi: 10.1038/s41467-021-21907-9 (PMC7955129; doi:10.1038/s41467-021-21907-9)
Supplement: Supplementary file 2 — Reporting Summary [file 41467_2021_21907_MOESM2_ESM.pdf]

nature research

Corresponding author(s): Ni Tang, Ailong Huang, Zeping Hu

Last updated by author(s): 21/12/20

Reporting Summary

Nature Research wishes to improve the reproducibility of the work that we publish. This form provides structure for consistency and transparency in reporting. For further information on Nature Research policies, see our Editorial Policies and the Editorial Policy Checklist.

Statistics

For all statistical analyses, confirm that the following items are present in the figure legend, table legend, main text, or Methods section.

n/a	Confirmed	
☐	☒	The exact sample size (*n*) for each experimental group/condition, given as a discrete number and unit of measurement
☐	☒	A statement on whether measurements were taken from distinct samples or whether the same sample was measured repeatedly
☐	☒	The statistical test(s) used AND whether they are one- or two-sided<br>*Only common tests should be described solely by name; describe more complex techniques in the Methods section.*
☒	☐	A description of all covariates tested
☐	☒	A description of any assumptions or corrections, such as tests of normality and adjustment for multiple comparisons
☐	☒	A full description of the statistical parameters including central tendency (e.g. means) or other basic estimates (e.g. regression coefficient) AND variation (e.g. standard deviation) or associated estimates of uncertainty (e.g. confidence intervals)
☐	☒	For null hypothesis testing, the test statistic (e.g. *F*, *t*, *r*) with confidence intervals, effect sizes, degrees of freedom and *P* value noted<br>*Give P values as exact values whenever suitable.*
☒	☐	For Bayesian analysis, information on the choice of priors and Markov chain Monte Carlo settings
☒	☐	For hierarchical and complex designs, identification of the appropriate level for tests and full reporting of outcomes
☐	☒	Estimates of effect sizes (e.g. Cohen's *d*, Pearson's *r*), indicating how they were calculated

Our web collection on statistics for biologists contains articles on many of the points above.

Software and code

Policy information about availability of computer code

Data collection	Targeted metabolomics data were collected with an AB QTRAP 6500+ triple quadrupole mass spectrometer (SCIEX, Framingham, MA), and untargeted metabolomics data were collected with a 6546 Q-TOF mass spectrometry equipped with a dual electrospray (ESI) ion source (Agilent Technologies, Santa Clara, CA).<br>Cytokines abundance in patients serum was measured by Bio-Plex Human Cytokine Screening Panel (48-Plex no. 12007283, Bio-Rad) on a Luminex 200 (Luminex Multiplexing Instrument, Merck Millipore). Cytokines produced by PBMCs were measured by MILLIPLEX MAP NonHuman Primate Cytokine Magnetic Bead Panel-Immunology Multiplex Assay (PRCYTOMAG-40K, Millipore USA).

Data analysis

SCIEX MultiQuant version 3.0.2
Agilent MassHunter Acquisition software version 10.1
Agilent Profinder software version 10.0
Agilent Pathways to PCDL software version B.08.00
Agilent Qualitative analysis version 10.0
SIMCA version 14.1
Rtsne version 0.15
mgcv version 1.8-31
Mfuzz version 2.44.0
igraph version 1.2.5
stat version 3.6.0
clusterProfiler version 3.12.0
GraphPad Prism version 8.2.1
Codes are available at https://github.com/amberxn/COVID19_NCOMMS.

For manuscripts utilizing custom algorithms or software that are central to the research but not yet described in published literature, software must be made available to editors and reviewers. We strongly encourage code deposition in a community repository (e.g. GitHub). See the Nature Research guidelines for submitting code & software for further information.

Data

Policy information about availability of data

All manuscripts must include a data availability statement. This statement should provide the following information, where applicable:

- Accession codes, unique identifiers, or web links for publicly available datasets
- A list of figures that have associated raw data
- A description of any restrictions on data availability

Clinical informations of COVID-19 patients, non-COVID-19 patients and healthy controls are included in Supplementary Data 1. Metabolomics raw mass spectrometry data are included in Supplementary Data 2. Normalized targeted and untargted metabolomics data are included in Supplemental Data 3. Cytokine and chemokine abundance are included in Supplemental Data 4. Data for each figure are included in Source Data.

Field-specific reporting

Please select the one below that is the best fit for your research. If you are not sure, read the appropriate sections before making your selection.

☒ Life sciences ☐ Behavioural & social sciences ☐ Ecological, evolutionary & environmental sciences

For a reference copy of the document with all sections, see nature.com/documents/nr-reporting-summary-flat.pdf

Life sciences study design

All studies must disclose on these points even when the disclosure is negative.

Sample size	No statistical methods were used to pre-determine sample size. For cross sectional analysis (n = 74), mean value of metabolites in each group were used and for longitudinal analysis (n = 7), mean value of metabolites in each time point were used for statistical tests with sample sizes taken into consideration. We rely on significant test to ensure the sample size is sufficient to draw conclusions.
Data exclusions	No data were excluded from the analysis.
Replication	Biological replications are as indicated in figure legends and methods. All attempts at replication were successful.
Randomization	For targeted and untargted metabolomics studies, serum metabolite samples were randomized into different sampling batches. We performed randomization of samples before acquisition on all of the other experiments.
Blinding	Each sample was labeled with an numeric ID whose annotation was kept blinded during data collection and analyses.

Reporting for specific materials, systems and methods

We require information from authors about some types of materials, experimental systems and methods used in many studies. Here, indicate whether each material, system or method listed is relevant to your study. If you are not sure if a list item applies to your research, read the appropriate section before selecting a response.

Materials & experimental systems

n/a	Involved in the study
☐	☒ Antibodies
☐	☒ Eukaryotic cell lines
☒	☐ Palaeontology and archaeology
☐	☒ Animals and other organisms
☐	☒ Human research participants
☒	☐ Clinical data
☒	☐ Dual use research of concern

Methods

n/a	Involved in the study
☒	☐ ChIP-seq
☒	☐ Flow cytometry
☒	☐ MRI-based neuroimaging

Antibodies

Antibodies used	Alkaline Phosphatase–conjugated Affinipure Goat Anti-Human IgG, Catalog number: SA00002-8, Proteintech.
Validation	The antibody was only used for the application as indicated and organisms verified by the manufactures.

Eukaryotic cell lines

Policy information about cell lines

Cell line source(s)	Vero E6 cells were purchased from ATCC.
Authentication	The African green monkey kidney epithelial Vero E6 is derived from ATCC, thus is already authenticated by STR profiling.
Mycoplasma contamination	The cell line was tested for mycoplasma contamination prior to commencement of experiments and was negative.
Commonly misidentified lines (See ICLAC register)	No commonly misidentified cell lines were used in this study.

Animals and other organisms

Policy information about studies involving animals; ARRIVE guidelines recommended for reporting animal research

Laboratory animals	Rhesus Macaque (n=1, mock-infected, female, age 5 years; n=1, SRAS-CoV-2-infected, female, age 5 years)
Wild animals	This study did not involve wild animals.
Field-collected samples	This study did not involve field-collected samples.
Ethics oversight	All animal procedures were approved by the Institutional Animal Care and Use Committee of Institute of Medical Biology, Chinese Academy of Medical Science.

Note that full information on the approval of the study protocol must also be provided in the manuscript.

Human research participants

Policy information about studies involving human research participants

Population characteristics	42 participants in this study were female and 39 male, with ages ranging from 0.66 to 80 yrs old and BMI 20.7–27.4 kg/m2. More information were provided in Methods and Supplemental Table 1.
Recruitment	Inclusion criteria were (1) SARS-COV-2 infection was confirmed by RT-PCR, (2) all the severe COVID-19 patients available were included and (3) mild cases , non-COVID-19 acute upper respiratory tract infection patients and healthy controls available with matched sex and BMI were included. Selection was based on availability and consent and there is no self-selection bias.
Ethics oversight	The study was approved by the Ethics Commission of Chongqing Medical University.

Note that full information on the approval of the study protocol must also be provided in the manuscript.
