# Supplementary material for: Integrated cytokine and metabolite analysis reveals immunometabolic reprogramming in COVID-19 patients with therapeutic implications
Source: Nat Commun. 2021 Mar 12;12:1618. doi: 10.1038/s41467-021-21907-9 (PMC7955129; doi:10.1038/s41467-021-21907-9)
Supplement: Supplementary file 3 — Description of Additional Supplementary Files [file 41467_2021_21907_MOESM3_ESM.pdf]

Description of Additional Supplementary Files

- **Supplementary Data 1** includes clinical information of COVID-19 patients, non-COVID-19 acute upper respiratory tract infection patients and healthy controls.
- **Supplementary Data 2** includes all raw metabolomics mass spectrometry data of serum COVID-19 patients, non-COVID-19 acute upper respiratory tract infection patients and healthy controls and those of PBMC pellets, culture media.
- **Supplementary Data 3** includes all normalized metabolomics data of serum from COVID-19 patients, non-COVID-19 acute upper respiratory tract infection patients and healthy controls and those of PBMC pellets, culture media, which were used for subsequent analyses.
- **Supplementary Data 4** includes cytokine abundance of serum from COVID-19 patients, non-COVID-19 acute upper respiratory tract infection patients and healthy controls and those of PBMC culture media.
